# NCOA7 inhibits renal cancer progression by inducing autophagy and lipid metabolism through V-ATPase interaction

**DOI:** 10.1038/s41420-025-02766-5

**Published:** 2025-10-21

**Authors:** Jianjun Wang, Huiwen Luo, Qingliu He, Honggang Shao, Xianfu Cai, Yuan Cao, Yaodong Wang, Decai Wang

**Affiliations:** 1 Department of Hepatobiliary Surgery, Mianyang Central Hospital, School of Medicine, University of Electronic Science and Technology of China, Mianyang, China; 2 NHC Key Laboratory of Nuclear Technology Medical Transformation, Mianyang Central Hospital, School of Medicine, University of Electronic Science and Technology of China, Mianyang, China; 3 Department of Urology, The Second Affiliated Hospital of Fujian Medical University, Quanzhou, China; 4 Department of Urology, Mianyang Central Hospital, School of Medicine, University of Electronic Science and Technology of China, Mianyang, China

**Keywords:** Urological cancer, Cancer

## Abstract

Clear cell renal cell carcinoma is the predominant pathological subtype of renal cell carcinoma, characterized by abnormal lipid metabolism. This study aimed to investigate the role of nuclear receptor co-activator 7 (NCOA7)-mediated autophagy in lipid metabolism in renal cancer and its effects on renal cancer progression. To this end, bioinformatic analysis was performed to analyze the prognostic significance of NCOA7 in renal cancer. Immunohistochemical staining and protein blotting were used to assess NCOA7 expression levels in clinical samples. Various assays, including scratch, Transwell, colony formation, and Oil Red O staining, were used to observe the effects of NCOA7 on the biological behavior and lipid metabolism of renal cancer cells. The interaction between NCOA7 and V-ATPase was investigated using immunoprecipitation. Additionally, NCOA7 expression was modulated in renal cancer cell lines transfected with LC3 dual-fluorescent virus to study its effects on lysosomal function, autophagy, and lipid metabolism. The effects of NCOA7 expression levels on subcutaneous tumors were investigated using a nude mouse xenograft model. Bioinformatics analysis showed that NCOA7 was expressed at low levels in renal cancer tissues and independently associated with the prognosis in patients with renal cancer. In vitro experiments showed that high NCOA7 expression inhibited the proliferation, migration, and invasion of renal cancer cells. NCOA7 promoted the fusion of lysosomes with autophagosomes through its interaction with V-ATPase. High expression of NCOA7 enhanced autophagy and lipid metabolism and inhibited the growth of renal cancer cells. In vivo experiments showed that high NCOA7 expression inhibited the growth of subcutaneous tumors in nude mice. Our study demonstrates that NCOA7 promotes autophagy and reduces lipid accumulation by binding to V-ATPase and ultimately inhibits the progression of renal cancer. These findings suggest that NCOA7 might be a potential target for drug intervention in clear cell renal carcinoma.

**Figure:**
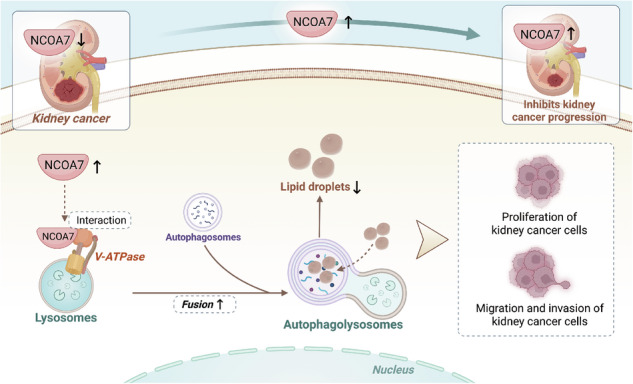
The schematic illustrates that NCOA7 upregulation interacts with V-ATPase to promote autophagosome–lysosome fusion, enhance autophagic degradation of lipid droplets, and thereby suppress proliferation, migration, and invasion of kidney cancer cells. Image created with BioRender.com, with permission.

The schematic illustrates that NCOA7 upregulation interacts with V-ATPase to promote autophagosome–lysosome fusion, enhance autophagic degradation of lipid droplets, and thereby suppress proliferation, migration, and invasion of kidney cancer cells. Image created with BioRender.com, with permission.

## Introduction

Renal cell carcinoma (RCC) is a significant urinary system malignancy, comprising between 80 and 90% of renal cancers [1]. Clear cell RCC (ccRCC) is the predominant subtype, making up about 70 to 80% of all renal cancers and is generally resistant to conventional radiotherapy and chemotherapy [2]. Early-stage renal cancer is typically treated with surgery, whereas advanced renal cancer is often managed with specific molecular targeted drugs and combined targeted immunotherapy. However, the limited tolerance of patients with advanced renal cancer to targeted drugs and their poor response to immunotherapy pose significant challenges in treatment. Therefore, there is an urgent need to further investigate the mechanisms underlying the pathogenesis and progression of ccRCC and identify new targets and options for treating advanced renal cancer.

ccRCC is characterized by significant abnormalities in lipid metabolism [3]. These metabolic disturbances lead to the accumulation of intracellular lipid droplets, a primary feature of clear cell carcinoma [4]. Recent research has increasingly focused on understanding the mechanisms underlying these lipid metabolism abnormalities and their biological roles in ccRCC development. Moreover, abnormal lipid metabolism contributes significantly to ccRCC progression by reducing endoplasmic reticulum stress, thereby maintaining the internal environment of ccRCC cells and promoting tumor growth [5]. Therefore, studying lipid metabolism represents a promising new direction in ccRCC research.

Autophagy is closely linked to intracellular metabolism [6–8]. Previous studies have shown the important role of autophagy in lipid metabolism and its role in degrading intracellular lipid droplets [9, 10]. Autophagosomes can encapsulate and degrade free fatty acids generated from lipid droplet catabolism, releasing them into the cytoplasm to regenerate ATP [11]. Autophagy is significantly inhibited in ccRCC, with the expression levels of autophagy-related genes showing a significant negative correlation with clinical stage and prognosis [12]. Another study found that autophagy in ccRCC initiated “lipid mobilization,” promoting the conversion of triglycerides to fatty acids and then degrading these lipids through uncoupling protein UCP1, which helped inhibit ccRCC progression [13]. While autophagy plays a role in promoting lipid degradation in ccRCC and is linked to prognosis, its specific regulatory mechanisms remain incompletely understood and require further investigation.

Nuclear receptor co-activator 7 (NCOA7) represents a class of proteins that binds to nuclear receptors in a ligand-dependent manner [14] and is present in almost all eukaryotic cells [15]. Initially identified as a protein associated with the estrogen receptor (ER) that enhances ER transcriptional activity, subsequent research has revealed that NCOA7 also facilitates the activation of other nuclear receptors, including the glucocorticoid receptor, thyroid hormone receptor B, peroxisome proliferator-activated receptor-Y, progesterone receptor, and retinoic acid receptor a [16]. Studies have shown that NCOA7 significantly promotes CYP1A1 expression in lung cancer cells compared to normal lung epithelial cells, promoting lung cancer progression [17]. In breast cancer cells, NCOA7 enhances *CYPIA1* transcription through coactivation of the AhR/AhRT complex [18], which in turn promotes breast cancer progression. Additionally, NCOA7 is involved in the proliferation of breast cancer cells [19], suggesting that inhibiting NCOA7 activity may help suppress breast cancer progression. Furthermore, NCOA7 has been shown to play a role in vitamin D transcriptional activation and vitamin D receptor complex formation [20], suggesting that NCOA7 could be a valuable indicator for assessing colorectal cancer risk [21].

The full-length NCOA7 consists of 942 complex acids, featuring a LysM structural domain at the carboxy terminus. TLDc proteins function as ligands for regulatory proteins of V-ATPase, playing roles in the regulation of extracellular plasmin secretion, vesicle acidification, and lysosomal activity. Research indicates that NCOA7 interacts with V-ATPase in renal tissue, and the absence of NCOA7 in mice leads to decreased V-ATPase expression and diminished uric acid acidification efficiency [22, 23]. Additionally, NCOA7 has been shown to interact with V-ATPase in the mouse brain and enhance lysosomal acidification in human glioma cell lines [24, 25]. Consequently, this study focused on exploring the role of NCOA7-mediated autophagy in lipid metabolism within renal cancer and its impact on the progression of the disease.

## Results

### Decreased NCOA7 expression in renal clear cell carcinoma is associated with specific clinical parameters and indicates an unfavorable prognosis in affected patients

**Fig. 1:**
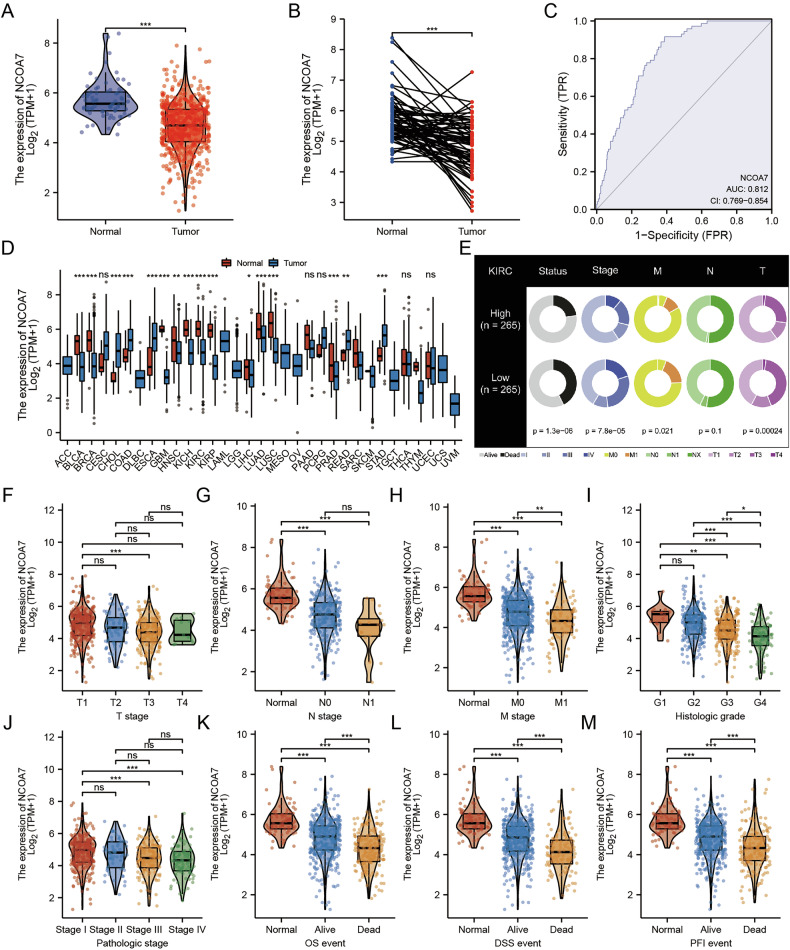
Decreased NCOA7 expression in renal clear cell carcinoma is associated with specific clinical parameters and indicates an unfavorable prognosis in affected patients. **A** Expression of NCOA7 in unpaired renal cancer tissues and normal tissues from the TCGA database. Compared to normal kidney tissues, the expression level of NCOA7 is significantly lower in unpaired renal cancer tissues. Normal: n = 72; Tumor: n = 532. **B** Expression of NCOA7 in paired renal cancer tissues and normal tissues from the TCGA database. Compared to normal kidney tissues, the expression level of NCOA7 is significantly lower in paired renal cancer tissues. Normal: n = 72; Tumor: n = 72. **C** ROC curve showing the area under the curve for NCOA7 as a biomarker for renal cancer. **D** Comparison of NCOA7 expression levels between different cancer tissues and normal tissues. Distinct NCOA7 expression disparities are noted between tumor and corresponding normal tissues. **E** Distribution of patients based on high and low NCOA7 expression levels. KIRC High: n = 265; KIRC Low: n = 265. **F**–**M** Relationship between NCOA7 expression levels and various clinical parameters. ns: not significant. ns: not significant. * *P* < 0.05, ** *P* < 0.01, *** *P* < 0.001. Data are presented as mean ± SD or median with interquartile range, as appropriate. Statistical analysis was performed using unpaired or paired Student’s t test, Wilcoxon rank-sum test, or Wilcoxon signed-rank test (two groups), and one-way ANOVA or Kruskal–Wallis test (multiple groups). The chi-square test was used for categorical variables. ROC analysis was applied to evaluate diagnostic performance. NCOA7 nuclear receptor co-activator 7.

Genes that were lowly expressed and exhibited tumor-suppressive effects in cancer patients were first screened from the TCGA database. Subsequently, univariate and multivariate Cox regression analyses were performed to exclude candidate genes that did not truly impact prognosis. Among the remaining candidates, we selected a previously unreported gene for functional experiments, ultimately identifying NCOA7 as our target gene. Our investigation revealed a marked downregulation of NCOA7 in ccRCC compared to that in normal kidney tissues, both in unpaired and paired tissues (Fig. 1A–B). The ROC curves also indicated that the expression level of NCOA7 could serve as a reliable diagnostic biomarker for ccRCC (Fig. 1C).

Regarding other malignancies, including breast cancer, cervical squamous cell carcinoma, cholangiocarcinoma, and colon adenocarcinoma, distinct NCOA7 expression disparities were noted between tumor and corresponding normal tissues (Fig. 1D). Between the distinct NCOA7 expression group, the results showed a significant difference in the distribution of patients with distinct status, pathologic stages, M stages and T stages between the two groups (Fig. 1E). Moreover, we discerned an incremental decrease in NCOA7 expression correlating with cancer progression (Fig. 1F–J). Patients with ccRCC who succumbed to the disease manifested decreased NCOA7 expression levels (Fig. 1K–M).

Given the aforementioned findings, we postulated a significant correlation between NCOA7 expression and survival in ccRCC patients. In line with this hypothesis, Kaplan-Meier analysis revealed that individuals with decreased NCOA7 levels faced more adverse outcomes concerning overall survival, disease-specific survival, and progression-free intervals (Supplementary Fig. 1A–C). Subsequent univariate and multivariate Cox regression analyses affirmed the role of NCOA7 expression as an autonomous predictor for survival outcomes in ccRCC patients (Supplementary Fig. 1D–E). Furthermore, the nomogram and calibration curve constructed using these indicators showed good performance (Supplementary Fig. 1F–G).

### NCOA7 inhibits the progression of ccRCC

**Fig. 2:**
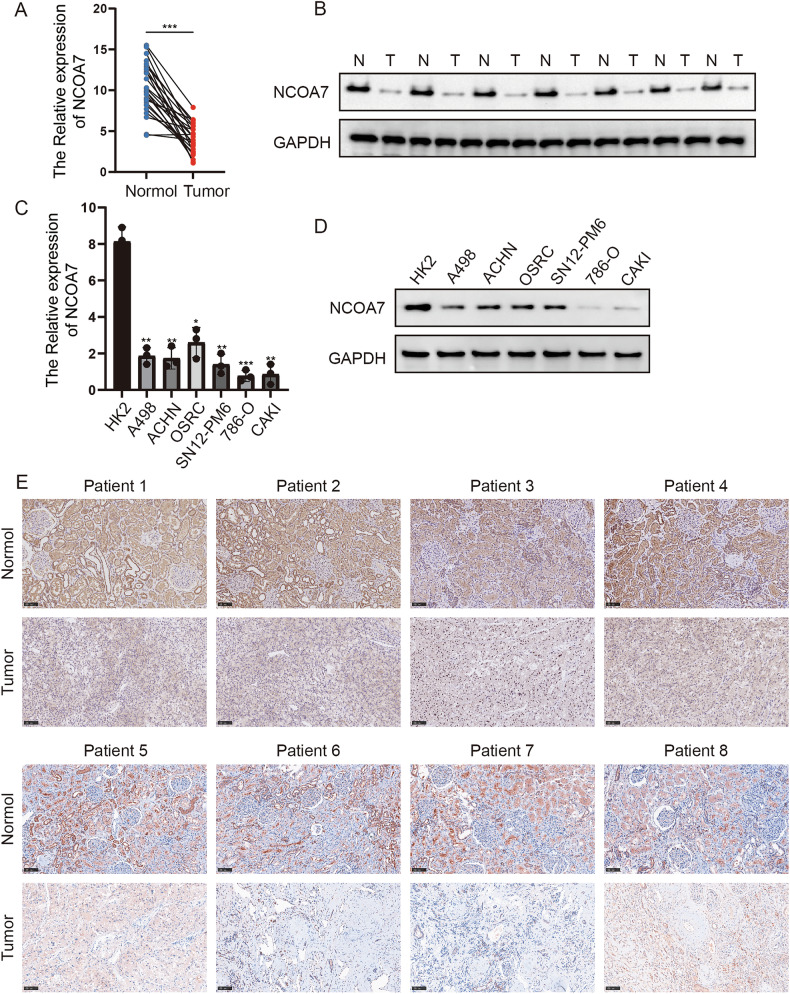
NCOA7 inhibits the progression of ccRCC. **A**–**B** Comparative mRNA and protein expression levels of NCOA7 in renal cancer tissues versus normal kidney tissues. Compared to normal kidney tissues, the expression level of NCOA7 is significantly lower in paired renal cancer tissues. Normal: n = 24; Tumor: n = 24. **C**–**D** Comparative mRNA and protein expression levels of NCOA7 in human normal renal tubular epithelial HK2 cells versus human renal cancer cell lines. n = 3. **E** Representative immunohistochemistry images depicting NCOA7 expression in human renal cancer tissues compared to normal kidney tissues. Scale bar: 100 μm. ns: not significant. *P < 0.05, **P < 0.01, *** *P* < 0.001. Data are presented as mean ± SD from at least three independent experiments. Statistical analysis was performed using paired Student’s t test (two-group paired samples) or Wilcoxon signed-rank test (**A**), and one-way ANOVA with Tukey’s post hoc test (multiple groups, **C**). ccRCC clear cell renal cell carcinoma, NCOA7 nuclear receptor co-activator 7.

To validate the bioinformatics analysis results, we examined NCOA7 protein and mRNA levels in ccRCC tissues. As shown in Fig. 2A–E, NCOA7 protein and mRNA levels were significantly reduced in ccRCC tissues. Similar findings were observed in ccRCC cell lines.

**Fig. 3:**
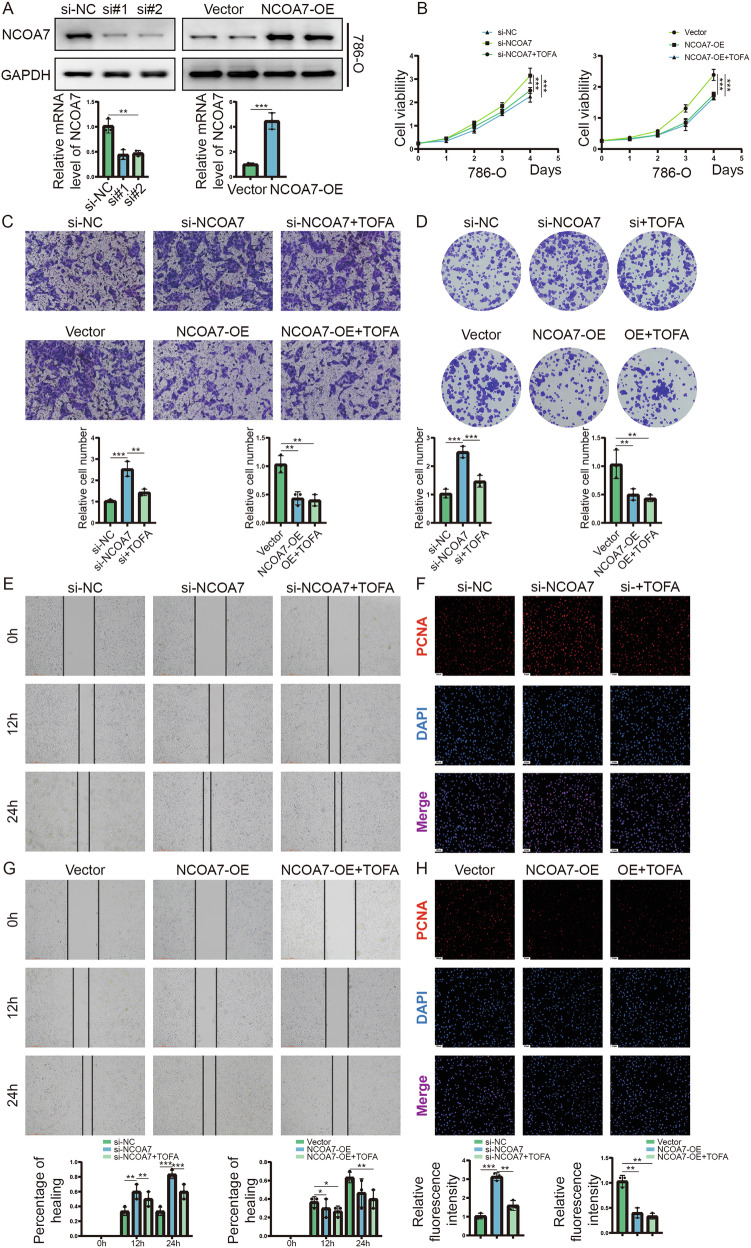
NCOA7 inhibits the progression of ccRCC. **A** Protein and mRNA levels of NCOA7 in 786-O cells with NCOA7 knockdown or overexpression. **B**–**H** MTT assay, Transwell assay, colony formation assay, scratch assay, and PCNA immunofluorescence assay to observe the effects of NCOA7 knockdown or overexpression on cell proliferation, migration, and invasion abilities in 786-O cell lines. Overall, inhibition of NCOA7 expression promotes the malignant behavior of renal cancer cells, whereas upregulation of NCOA7 expression suppresses their biological behavior. Scale bar: 100 μm. ns: not significant. *P < 0.05, **P < 0.01, ***P < 0.001. Data are presented as mean ± SD from at least three independent experiments. Statistical significance was determined using unpaired Student’s t test (two groups), one-way ANOVA with Tukey’s post hoc test (multiple groups), or two-way ANOVA for wound-healing assays with time-course data. ccRCC clear cell renal cell carcinoma, NCOA7 nuclear receptor co-activator 7.

The notable differential expression of NCOA7 in ccRCC suggests its potential influence on the biological functions of this cancer type. To investigate this, we created stable 786-O and CAKI cell lines with either overexpression or knockdown of NCOA7 using lentiviral vectors and shRNA (Fig. 3A, Supplementary Fig. 2A). The MTT assay demonstrated that cells overexpressing NCOA7 exhibited reduced proliferation compared to control cells; however, knockdown of NCOA7 significantly enhanced cell proliferation (Fig. 3B, Supplementary Fig. 2B). The Transwell assay showed that NCOA7 overexpression significantly impaired cell migration and invasion capabilities (Fig. 3C, Supplementary Fig. 2C). Furthermore, NCOA7 overexpression diminished the colony-forming ability of the cells in vitro (Fig. 3D, Supplementary Fig. 2D). Wound-healing assays indicated that high NCOA7 expression significantly suppressed the migratory ability of ccRCC cells (Fig. 3E, Supplementary Fig. 2E). In contrast, knockdown of NCOA7 significantly enhanced the biological behavior of renal cancer cells (Fig. 3C–E, Supplementary Fig. 2C–E). To further assess the impact of NCOA7 overexpression on ccRCC cell proliferation, we measured PCNA expression via immunofluorescence staining. As shown in Fig. 3F and Supplementary Fig. 2F, PCNA expression was markedly lower in the NCOA7 overexpression group than in vector group, whereas it was markedly higher in the NCOA7 knockdown group than in control group, indicating a significant reduction in ccRCC cell proliferation. These findings suggest that NCOA7 functions as a tumor suppressor, inhibiting the progression of ccRCC.

### NCOA7 plays a role in ccRCC lipid accumulation

**Fig. 4:**
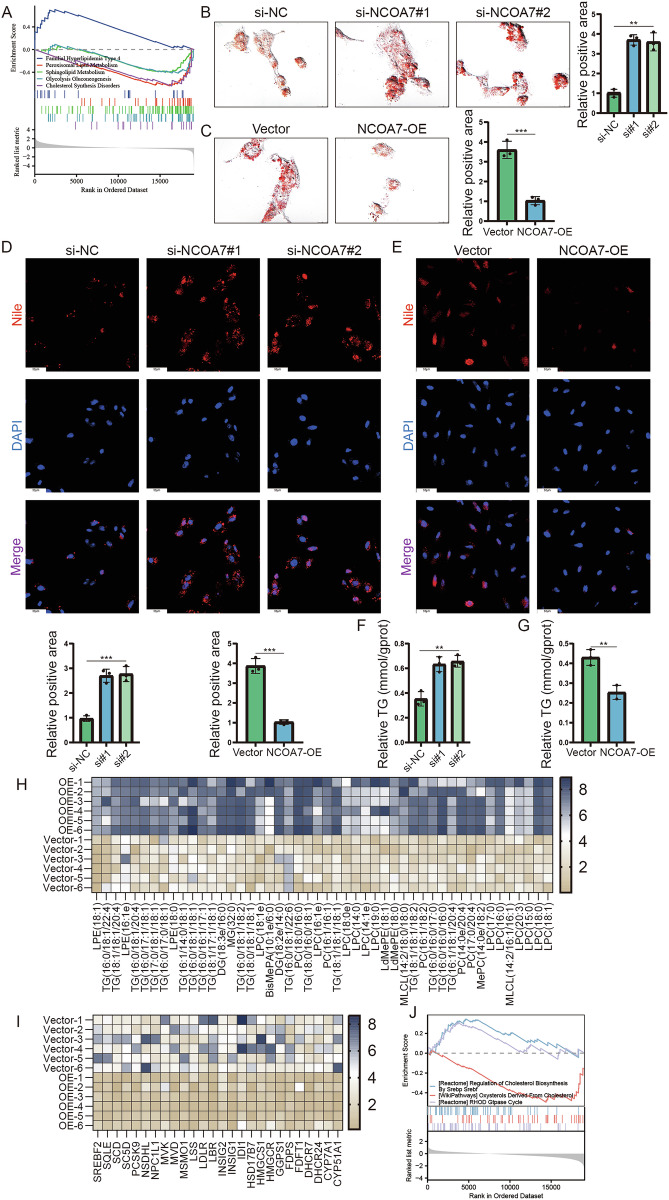
NCOA7 plays a role in ccRCC lipid accumulation. **A** GSEA results showing the correlation between lipid/lipoprotein metabolism and NCOA7 mRNA levels in ccRCC. **B** Microscopic images of Oil Red O staining in 786-O cells with NCOA7 knockdown. Scale bar: 25 μm. n = 3. **C** Microscopic images of Oil Red O staining in cell lines overexpressing NCOA7 compared to the negative control. Scale bar: 25 μm. n = 3. **D**, **E** Representative images of immunofluorescence staining for lipids with Nile red in cells with either NCOA7 knockdown or overexpression. Scale bar: 50 μm. n = 3. **F**, **G** Relative TG (mmol/gprot) levels in cells with NCOA7 overexpression and knockdown assessed by a triglyceride assay kit. n = 3. **H**, **I** Lipid metabolism and cholesterol metabolomics analysis of 786-O cells with control or overexpression of NCOA7 (n = 6). **J** GSEA results showing the correlation between cholesterol metabolism and NCOA7 mRNA levels in ccRCC. ns: not significant. *P < 0.05, **P < 0.01, ***P < 0.001. Data are presented as mean ± SD from at least three independent experiments. Statistical significance was determined using unpaired Student’s t test (two groups), one-way ANOVA with Tukey’s post hoc test (multiple groups, **B**–**G**). GSEA was performed to identify enriched pathways, and significance was assessed using Kolmogorov–Smirnov statistics with false discovery rate (FDR) adjustment. ccRCC clear cell renal cell carcinoma, NCOA7 nuclear receptor co-activator 7.

ccRCC is a typical example of metabolic reprogramming, with aberrant lipid accumulation being a prominent feature. GSEA showed that NCOA7 was associated with lipid metabolism in ccRCC, potentially regulating lipolytic metabolic processes and lipase activity (Fig. 4A). To investigate lipid browning in tumor cells, we knocked down or overexpressed NCOA7 using a lentivirus or plasmid in 786-O and CAKI renal carcinoma cells. Oil Red O staining showed that NCOA7 knockdown significantly increased lipid deposition in renal carcinoma cells, whereas overexpression of NCOA7 eliminated abnormal lipid accumulation (Fig. 4B–C, Supplementary Fig. 3A–B). Additionally, Nile Red staining confirmed that NCOA7 knockdown significantly increased lipid deposition in renal cancer cells compared to controls, whereas NCOA7 overexpression effectively reduced lipid droplet deposition and the relative diameters of lipid droplets (Fig. 4D–E, Supplementary Fig. 3C–D). Triglyceride content measurements using a triglyceride assay kit supported these findings: NCOA7 overexpressing cell lines exhibited relatively low triglyceride content, whereas NCOA7 knockdown cells had a higher triglyceride content (Fig. 4F–G, Supplementary Fig. 3E–F). Furthermore, we employed TOFA, an inhibitor of lipid biosynthesis, to investigate whether NCOA7 influences the biological behavior of renal cancer cells by regulating lipid metabolism. The results showed that TOFA could reverse the biological effects induced by NCOA7 knockdown (Fig. 3B-H, Supplementary Fig. 2B–H), and it also synergistically enhanced the inhibitory effects of NCOA7 overexpression on renal cancer cell behavior (Fig. 3B–H, Supplementary Fig. 2B–H).

We focused on examining the changes in key lipid species and cholesterol regulated by NCOA7 in tumor cells. Lipidomic analyses revealed that NCOA7 overexpression significantly reduced the accumulation of triglycerides and cholesterol esters in RCC cells, which is consistent with the findings reported by Harvey et al [26] (Fig. 4H–I). GSEA analysis further confirmed that NCOA7 regulates not only lipid metabolism but also cholesterol metabolism (Fig. 4J). In summary, NCOA7 is a critical factor in regulating lipid accumulation in ccRCC and affects the proliferation and invasiveness of renal cancer cells by modulating lipid and cholesterol metabolism.

### NCOA7 mediates cellular lipid accumulation in tumors by inducing autophagic flow

**Fig. 5:**
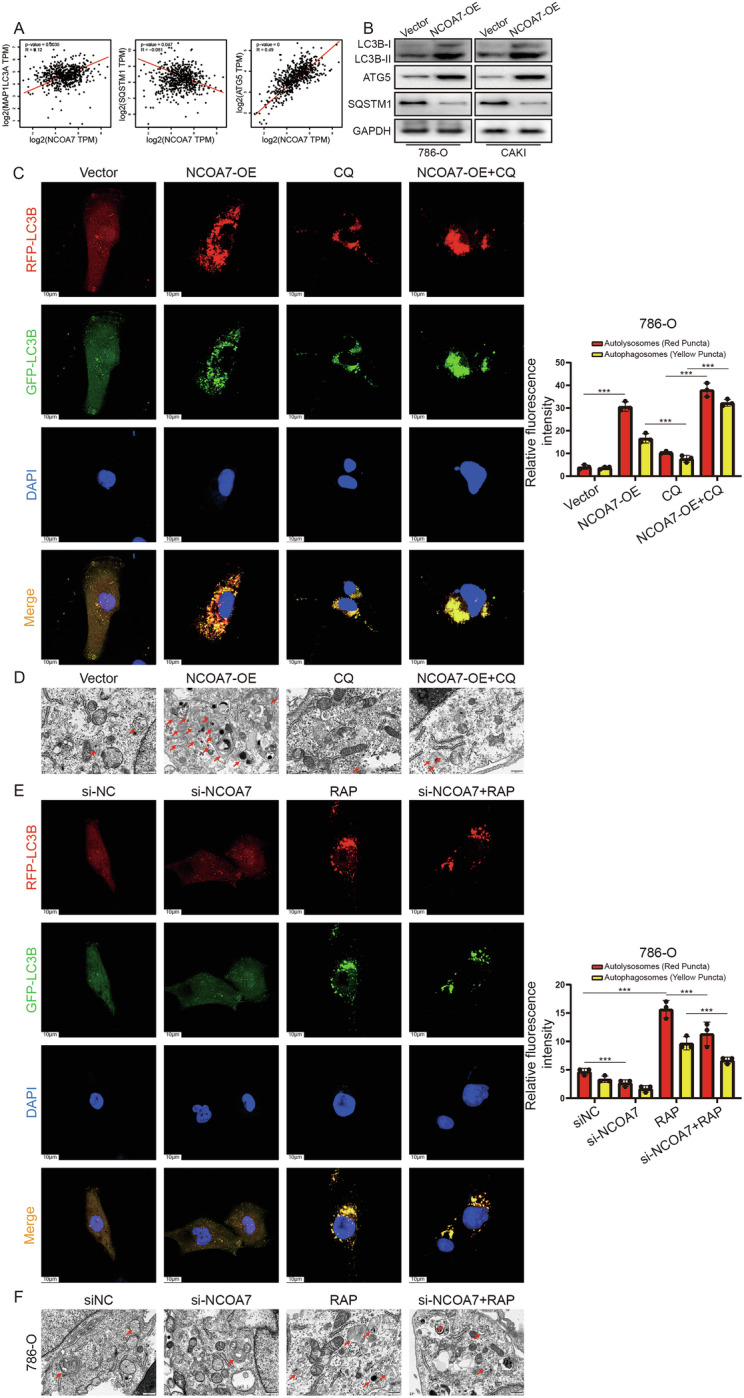
NCOA7 mediates cellular lipid accumulation in tumors by inducing autophagic flow. **A** Correlation between NCOA7 expression levels and autophagy-related proteins (MAP1LC3A, SQSTM1, ATG5). **B** Effect of NCOA7 overexpression on the autophagy-related protein LC3, ATG5, and SQSTM1. **C** Confocal microscopy images showing the impact of different treatments on autophagosomes. Overexpression of NCOA7 leads to increased autophagic flux, indicated by the presence of red puncta similar to RAP treatment, whereas CQ inhibits autophagy induced by NCOA7. Scale bar: 10 μm. n = 3. **D** Electron microscopy images showing the impact of different treatments on autophagosomes. Overexpression of NCOA7 significantly increases the number of autophagosomes (indicated with red arrows). In NCOA7-overexpressing cells, typical features of enhanced autophagy, including increased numbers of autophagosomes and autolysosomes, as well as evidence of accelerated lipid droplet turnover, were observed. These images provide morphological support for the role of NCOA7 in promoting autophagy and lipid metabolism. Scale bar: 500 nm. **E** Confocal microscopy images showing the impact of different treatments on autophagosomes. NCOA7 knockdown resulted in reduced autophagic flux. Scale bar: 10 μm. n = 3. **F** Electron microscopy images showing the impact of different treatments on autophagosomes. Inhibition of NCOA7 expression markedly reduces the number of autophagosomes. Scale bar: 500 nm. n = 3. ns: not significant. *P < 0.05, **P < 0.01, ***P < 0.001. Data are presented as mean ± SD from at least three independent experiments. Statistical analysis was performed using Pearson correlation (**A**), one-way ANOVA with Tukey’s post hoc test (**C**, **E**), and Student’s t test where appropriate. ccRCC clear cell renal cell carcinoma, NCOA7 nuclear receptor co-activator 7.

Many studies have shown that autophagy is associated with lipid droplet metabolism [27–29]. GEPIA analysis demonstrated that NCOA7 is closely associated with autophagy-related molecules in ccRCC (Fig. 5A). Cells with high NCOA7 expression showed higher levels of LC2B-II than that of LC3B-I (Fig. 5B). Furthermore, transient expression of NCOA7 resulted in increased ATG5 levels (Fig. 5B). We also observed that NCOA7 expression significantly decreased SQSTM1 (p62) levels, suggesting that NCOA7 promotes autophagic flux.

Next, we investigated the effects of NCOA7 overexpression on autophagosome formation. 786-O and CAKI cells were transfected with plasmids and mRFP-GFP-LC3B reporter constructs and treated with Rapamycin (RAP) or Chloroquine (CQ). GFP fluorescence is quenched in acidic conditions; hence, only red fluorescence was detected when autophagosomes fuse with lysosomes. Overexpression of NCOA7 led to increased autophagic flux, indicated by the presence of red puncta similar to RAP treatment, whereas CQ inhibited autophagy induced by NCOA7 (Fig. 5C, Supplementary Fig. 4A). In contrast, NCOA7 knockdown resulted in reduced autophagic flux (Fig. 5E, Supplementary Fig. 4C). Scanning electron microscopy further confirmed that NCOA7 promoted the formation of autophagic vesicles (Fig. 5D, F, Supplementary Fig. 4B, D).

**Fig. 6:**
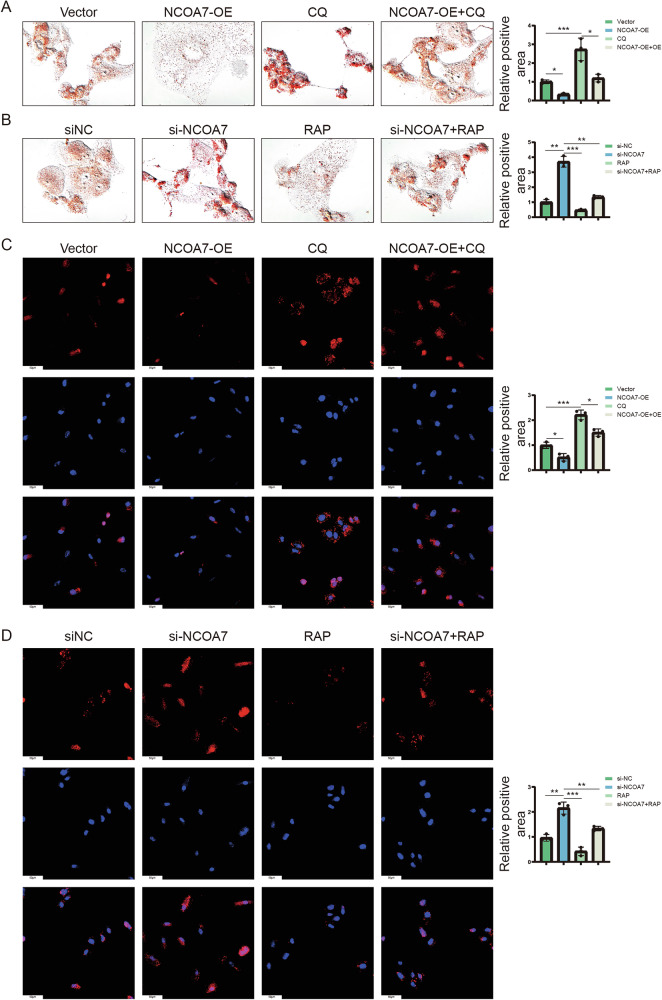
NCOA7 mediates cellular lipid accumulation in tumors by inducing autophagic flow. **A**, **B** Photomicrographs of Oil Red O staining of the 786-O cell lines. Scale bar: 25 μm. n = 3. **C**, **D** Representative photographs of immunofluorescence staining for lipids with Nile red in the 786-O cell lines. Scale bar: 50 μm. n = 3. ns: not significant. *P < 0.05, **P < 0.01, ***P < 0.001. Data are presented as mean ± SD from at least three independent experiments. Statistical significance was determined using one-way ANOVA followed by Tukey’s post hoc test for multiple group comparisons. NCOA7, nuclear receptor co-activator 7.

Subsequently, we used Oil Red and Nile staining to investigate the effects of NCOA7 expression and autophagy changes on lipid metabolism in renal cancer cells. The results showed that the reduced lipid accumulation observed in NCOA7 overexpressing cells was altered by the inhibition of autophagy (Fig. 6A, C, Supplementary Fig. 5A, C). In contrast, the increased lipid accumulation observed in NCOA7 knockdown cells was reversed by increased autophagy (Fig. 6B, D, Supplementary Fig. 5B, D). These findings indicate that NCOA7 mediates lipid depletion in tumor cells by inducing autophagy.

### Interaction between NCOA7 and v-ATPase is required for NCOA7 accumulation in autophagosomes

**Fig. 7:**
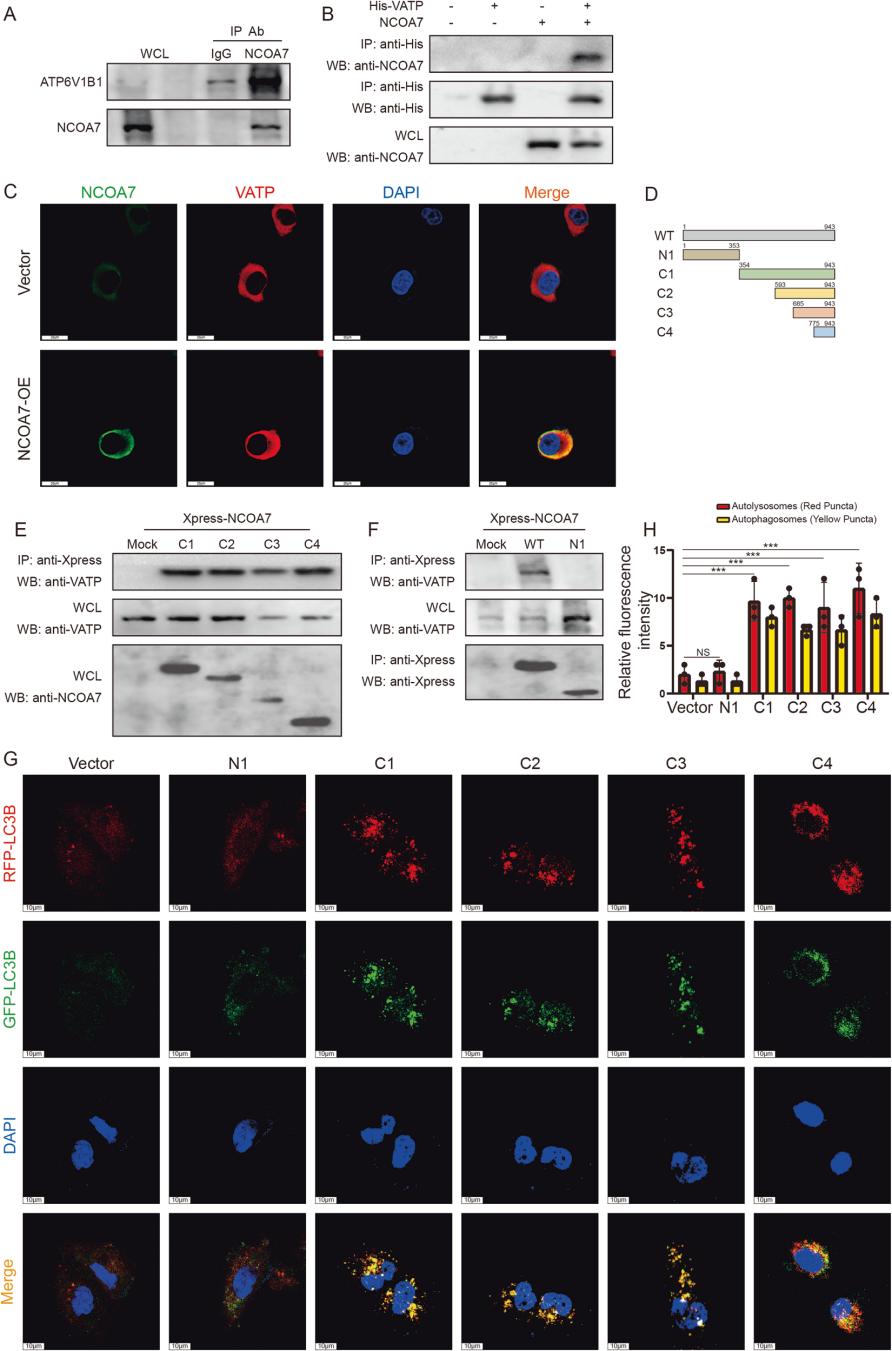
The interaction between NCOA7 and v-ATPase is required for NCOA7 to accumulate autophagosomes. **A** Interaction between NCOA7 and ATP6V1B1. 786-O cells are immunoprecipitated using either normal IgG or an anti-NCOA7 antibody, followed by immunoblotting with an anti-ATP6V1B1 antibody (upper panels) or an anti-NCOA7 antibody (lower panel). WCL refers to whole cell lysates. **B** Interaction between NCOA7 and ATP6V1B1. 786-O cells are transfected with HA-tagged NCOA7, His-tagged ATP6V1B1 (His-ATP6V1B1), or both. ATP6V1B1 protein is immunopurified using an anti-His antibody, and the immunoprecipitates are immunoblotted with an anti-NCOA7 antibody. **C** Colocalization of NCOA7 and ATP6V1B1. Scale bar: 20 μm. **D** Schematic diagram of NCOA7 and its deletion mutants (N1, C1 to C4). Numbers correspond to amino acid sequences. **E** Identification of the NCOA7 region required for V-ATPase interaction. 786-O cells are transfected with Xpress-tagged NCOA7 mutants (C1, C2, C3, C4), and Xpress-NCOA7 mutants are immunopurified using an anti-Xpress antibody. The immunoprecipitates are probed with an anti-ATP6V1B1 antibody. **F** 786-O cells are transfected with either wild-type Xpress-NCOA7 (WT) or Xpress-tagged NCOA7 N1. Xpress-NCOA7 proteins are immunopurified using an anti-Xpress antibody, and immunoprecipitates are immunoblotted with an anti-ATP6V1B1 antibody. **G**, **H** The C-terminal domain of NCOA7 is required for autophagosome formation. NCOA7 deletion mutants were expressed in GFP-RFP-LC3B cells. Scale bar: 20 μm. ns: not significant. *P < 0.05, **P < 0.01, ***P < 0.001. Data are presented as mean ± SD from at least three independent experiments or independent mice per group. Statistical analysis was performed using two-way ANOVA for tumor growth curves, and unpaired Student’s t test or one-way ANOVA with Tukey’s post hoc test for group comparisons (tumor weight, staining quantification, and protein expression). NCOA7 nuclear receptor co-activator 7.

Previous studies have shown that NCOA7, a member of the protein family with a conserved “TLDc” structural domain, interacts with V-ATPase and is involved in extracellular proton secretion, vesicle acidification, and lysosomal function [22, 23]. We investigated whether the TLDc domain of NCOA7 interacts with V-ATPase, affects lysosomal binding to autophagosomes, and regulates autophagic flow. We identified ATP6V1B1 as a potential binding partner using the STRING v9.1 protein-protein interaction network analysis tool. Immunoprecipitation assays showed the interaction between NCOA7 and ATP6V1B1 (Fig. 7A, Supplementary Fig. 6A). Furthermore, this interaction was confirmed by transient expression of NCOA7 and His-ATP6V1B1 (Fig. 7B, Supplementary Fig. 6B). Subcellular localization studies using confocal microscopy showed significant overlap between NCOA7 and ATP6V1B1 (Fig. 7C, Supplementary Fig. 6C).

To elucidate the molecular interaction between NCOA7 and V-ATPase, we divided the interaction site into four segments: (1) N-terminal NCOA7 (2-353), which includes a LysM structural domain of unknown function; [2] C1 (354-943); and (3) C2 NCOA7 (593-943); C3 (685-943); and C4 (775-943) (Fig. 7D). 786-O and CAKI cells were transfected with His-tagged NCOA7 mutants (C1, C2, C3 and N4), and these His-NCOA7 mutants were immunopurified using an anti-His antibody. Immunoprecipitation with an anti-ATP6V1B1 antibody revealed that only the C-terminal region of NCOA7 (775-943) interacted with the ATP6V1B1 subunit, while the N-terminal and intermediate regions did not (Fig. 7E, Supplementary Fig. 6D). In addition, we examined whether ATPV1B1 is associated with the NCOA7 N-terminal mutant. Immunoprecipitation showed that the NCOA7 N-terminus did not interact with ATPV1B1 (Fig. 7F, Supplementary Fig. 6E). These findings indicate that the C-terminus of NCOA7 is essential for binding to V-ATPase.

Next, we investigated the effects of truncated NCOA7 mutants on autophagosome formation. The results showed that the NCOA7 C-terminal mutant (C1-C4), which interacts with V-ATPase, induced autophagosome accumulation (Fig. 7G–H, Supplementary Fig. 6F–G). In contrast, N-terminal and intermediate NCOA7 mutants did not induce autophagosome accumulation or increase LC3B-II levels (Fig. 7G–H, Supplementary Fig. 6F–G). These results suggest that the C-terminal structural domain of NCOA7 is required for autophagosome formation.

### Autophagy mediates the biological effects of NCOA7 on renal cancer cells

**Fig. 8:**
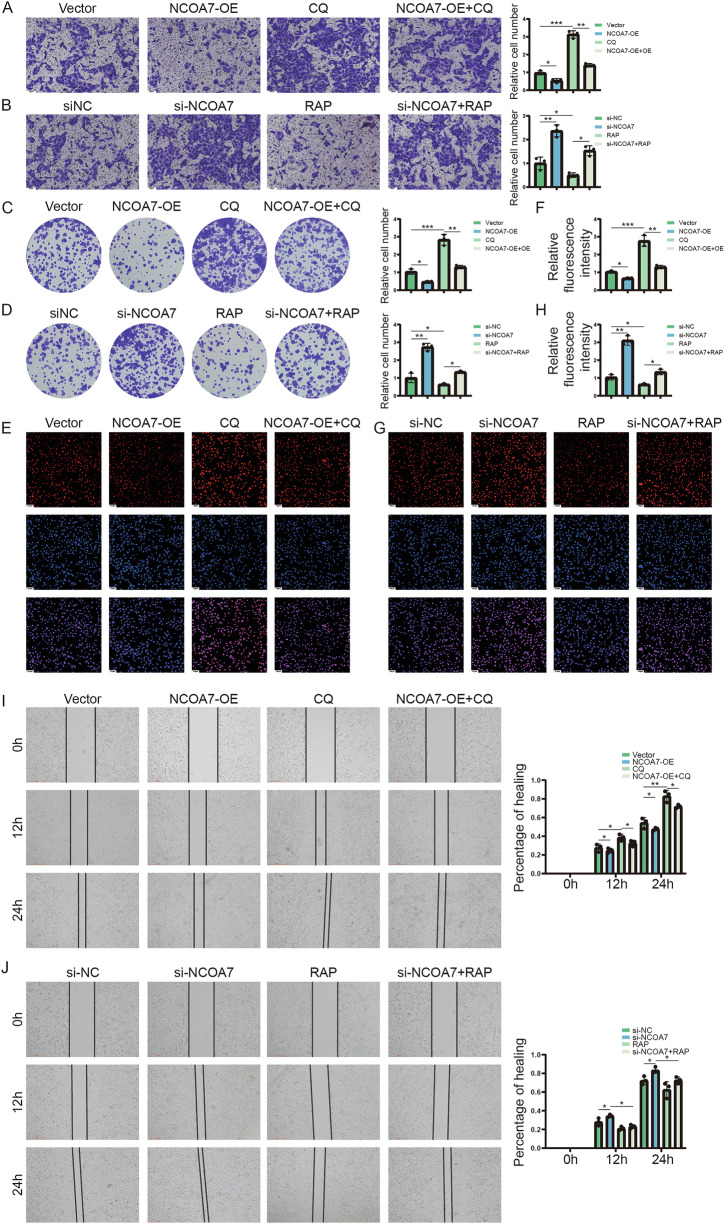
Autophagy mediates the biological effects of NCOA7 on renal cancer cells. **A**, **B** Transwell assay is used to observe the migratory ability of NCOA7 overexpressed or knockdown 786-O cell lines. Scale bar: 100 μm. n = 3. **C**, **D** Colony formation assay is used to observe the proliferation ability of NCOA7 overexpressed or knockdown 786-O cell lines. n = 3. **E**–**H** PCNA immunofluorescence assay was used to observe the proliferation ability of NCOA7 overexpressed or knockdown 786-O cell lines under different treatments. Scale bar: 20 μm. n = 3. **I**, **J** Scratch assay is used to observe the proliferation ability NCOA7 overexpressed or knockdown 786-O cell lines. n = 3. ns: not significant. *P < 0.05, **P < 0.01, ***P < 0.001. Data are presented as mean ± SD from at least three independent experiments or independent mice per group. Statistical analysis was performed using two-way ANOVA for tumor growth curves, and unpaired Student’s t test or one-way ANOVA with Tukey’s post hoc test for group comparisons (tumor weight, staining quantification, and protein expression). NCOA7 nuclear receptor co-activator 7.

Since NCOA7 enhances autophagy and reduces tumor cell lipid accumulation by binding to V-ATPase, we further investigated its biological effects in renal cancer cells. We performed Transwell invasion assays, revealing that inhibiting autophagy reversed the effects of high NCOA7 expression on migration (Fig. 8A–B, Supplementary Fig. 7A–B). Colony formation and immunofluorescence staining of PCNA showed that autophagy inhibition reversed the suppressive effects of high NCOA7 expression on cell migration and colony formation (Fig. 8C–F, Supplementary Fig. 7C–F). In addition, in vitro wound-healing assays indicated that autophagy inhibition could counteract the growth-promoting effects of high NCOA7 expression (Fig. 8G–H, Supplementary Fig. 7G–H). These results indicate that NCOA7 suppresses renal cancer cell activity through its role in autophagy.

### In vivo overexpression of NCOA7 inhibited the progression of ccRCC

**Fig. 9:**
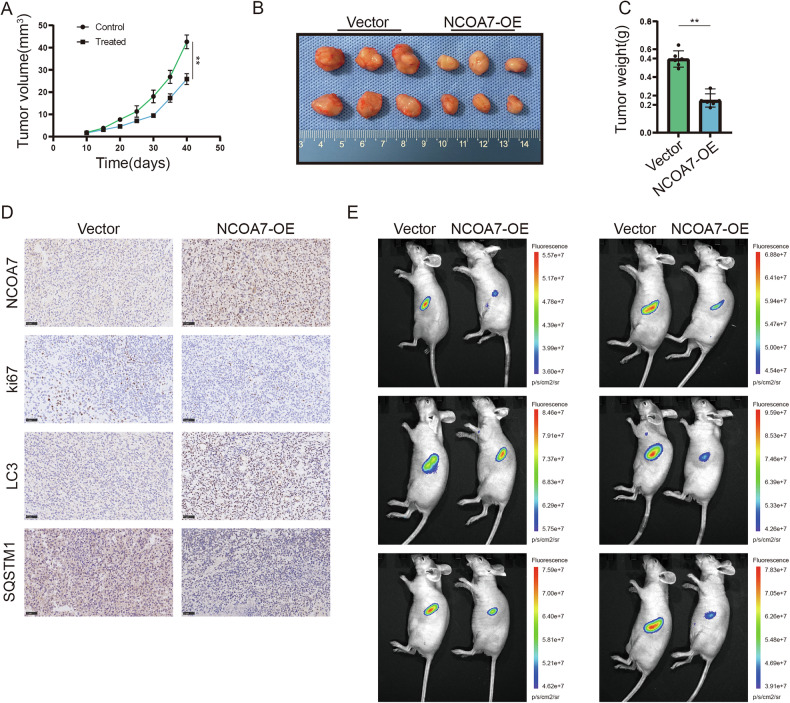
In vivo overexpression of NCOA7 inhibits the progression of ccRCC. **A** Quantification of tumor volumes. 786-O cells with stable overexpression of NCOA7 are injected into nude mice. Tumor size is measured every 5 days. n = 6. **B** Images of tumors dissected from the mice are shown. Vector: n = 6; NCOA7-overexpressing: n = 6. **C** At the conclusion of the experiment, tumors are weighed post-resection. n = 6. **D** Immunohistochemical (IHC) staining of tumor xenografts from both the NCOA7-overexpressing and control groups were conducted. Scale bar: 50 μm. **E** Living fluorescence images of the metastasis model in the NCOA7 overexpression and control groups. ns: not significant. *P < 0.05, **P < 0.01, ***P < 0.001. Data are presented as mean ± SD (n = indicated mice per group). Statistical analysis was performed using two-way ANOVA for tumor growth curves (**A**) and unpaired Student’s t test for tumor weight and quantitative staining results (**C**, **D**, **E**). NCOA7, nuclear receptor co-activator 7.

To further explore the role of NCOA7, we used an animal model. We injected 786-O cells overexpressing NCOA7 into nude mice to construct a xenograft tumor model. Tumor size was measured every 5 days, with the final measurement taken at 40 days. The results showed that tumors in the NCOA7 overexpression group had significantly reduced volume and weight (Fig. 9A–C).

We also evaluated autophagy levels and tumor malignancy using IHC. NCOA7 overexpression promoted the upregulation of LC3-II and the downregulation of SQSTM1, whereas the tumor malignancy marker Ki67 was significantly reduced (Fig. 9D). Additionally, using the tail vein metastasis model, it was evident that NCOA7 overexpression substantially decreased liver metastasis of tumor cells (Fig. 9E). Collectively, these results indicate that NCOA7 plays a critical role in curbing the progression of ccRCC.

## Discussion

Lipid accumulation is a key feature of ccRCC metabolic reprogramming, and studies have shown that lipid accumulation promotes tumor cell growth and invasion [3, 30, 31]. However, the mechanisms by which lipid metabolism affects the biological behavior of ccRCC cells are not fully understood. The NCOA family consists of transcriptional coactivators involved in diverse cellular processes, including metabolism, proliferation, and tumor progression [32]. We identified NCOA7 as a key gene involved in ccRCC metabolic reprogramming, mainly regulating autophagy and lipid metabolism. This study suggests that NCOA7 promotes autophagy in renal cancer cells by binding to V-ATPase, which in turn improves lipid metabolism and suppresses tumor cell behavior. This finding may provide potential targets for future therapies. Lipid droplets are composed of a neutral lipid core containing triglycerides and cholesteryl esters [33]. During starvation, Lipid droplet catabolism produces free fatty acids, which are then translocated to mitochondria for β-oxidation via the autophagy/lysosomal pathway [34]. In the present study, we found that autophagy was inhibited in ccRCC and that lipid droplets accumulated. The role of autophagy in mediating lipolysis to selectively convert lipid droplets into FFAs in ccRCC cells remains unclear. Previous studies have identified a conserved “TLDc” structural domain that interacts with the proton pump V-ATPase [35]. In addition, NCOA7, known to be regulated by V-ATPase in the brain, plays a role in regulating lysosomal function, neuronal connectivity, and behavior [24]. The present study demonstrates that restoring NCOA7 expression in ccRCC cells significantly activates lipid autophagy, leading to increased lipolysis.

Lipoautophagy refers to a specific type of autophagy where lipid droplets are selectively targeted for lysosomal degradation [10]. Autophagy has emerged as a promising therapeutic target for a wide range of diseases [36]. Autophagic flux was assessed by concurrently analyzing the levels of LC3-II and LC3- I in the autophagosome membrane and measuring the levels of SQSTM1. I and SQSTM1 levels were measured to assess the autophagic flux. The combination of an increased LC3-II/I ratio and decreased SQSTM1 levels was consistent with an overall increase in autophagic flux. In this study, we found that NCOA7 overexpression triggered autophagy, increased autophagy-associated membrane LC3 synthesis, and partially promoted autophagic flux. In addition to molecular and biochemical assays, ultrastructural evidence further supported our conclusions. TEM images of NCOA7-overexpressing renal cancer cells revealed typical autophagic features, including an increased number of autophagosomes and autolysosomes, accompanied by a reduction in intact lipid droplets. These morphological observations are consistent with the hypothesis that NCOA7 enhances autophagy and facilitates lipid droplet turnover, thereby accelerating lipid metabolism. Furthermore, an inverse correlation was observed between abnormal lipid accumulation and autophagy. Interestingly, intracellular lipids levels increased when NCOA7-induced autophagosome formation was restricted. Mul1 promoted autophagic flux to facilitate the degradation of SQSTM1-associated protein aggregates and ADFP-associated lipid droplets, which in turn inhibits ccRCC cell growth and migration [37]. Additionally, NNT, regulated by HIF2α, significantly activates lipid browning, leading to depletion in tumor cells and progression of ccRCC [38]. Our findings also showed that overexpression of NCOA7 reduced intracellular lipid accumulation and inhibited ccRCC cell growth and migration.

Studies have shown that the interferon-inducible short isoform of human NCOA7 inhibits viral entry, interacts with vesicular ATPase, and promotes endolysosomal vesicle acidification and lysosomal protease activity [39]. Both NCOA7 and its antisense transcript (NCOA7-AS) interact with several subunits of the vesicular proton pump, V-ATPase, leading to increased acidification of the endolysosomal system and reduced viral entry into host cells via the endosomal pathway [40]. NCOA7 interacts with the cytoplasmic domain of V-ATPase in the brain, and its absence results in altered neuronal development and defective lysosome formation and function [24]. A recent report suggested that the NCOA7 isoform 4 (NCOA7iso4) interacts with endolysosomal vesicular V-ATPase to increase its cleavage activity, significantly impairing vesicular stomatitis virus glycoprotein entry [41, 42]. NCOA7 interacts with V-ATPase, promoting cytoplasmic vesicle acidification, lysosomal protease activity, and the degradation of endocytosed antigens [25]. NCOA7 is involved in intracellular function and urinary acidification in mice, possibly by regulating the abundance of V-ATPase and other key acid-base regulators in the renal medulla [23]. Recent studies have revealed that multiple members of the NCOA family may play important roles in RCC. Notably, NCOA2 has been reported as a potential biomarker in ccRCC. A study by Chen et al. (2023)demonstrated that NCOA2 expression was significantly downregulated in ccRCC tissues, likely owing to promoter hypermethylation [43]. Higher NCOA2 expression was associated with improved overall survival and a more favorable immune microenvironment, suggesting its involvement in tumor immune regulation and potential prognostic value. In addition, NCOA1 has also been implicated in ccRCC. Jia et al. showed that elevated NCOA1 expression correlated with better overall and disease-free survival, supporting its role as an independent prognostic factor in RCC [44]. Beyond expression profiles, structural alterations involving NCOA family genes have also been linked to renal tumors. Argani et al. (2018) reported two cases of primary renal sarcoma characterized by MEIS1-NCOA2 gene fusions, defining a unique renal sarcoma subtype with distinct histological features and aggressive potential [45]. Our study identified that NCOA7 contains a “TLDc” structural domain that interacts with V-ATPase. This interaction impacts autophagy in lysosomes, which in turn affects lipid autophagy and, ultimately, renal cancer cell development.

## Conclusion

Overall, our results suggest that enhancing the expression level of NCOA7, promoting autophagic flow, and inhibiting lipid accumulation may be effective measures for the treatment of renal cancer.

## Materials and methods

### Bioinformatic data collection and processing

RNA-sequencing data pertaining to ccRCC patients was acquired from The Cancer Genome Atlas (TCGA) database in raw count format. Subsequently, the original data was processed using R software to convert them into Transcripts Per Kilobase of exon model per Million mapped reads (TPM). Concurrently, pertinent clinical information, encompassing cancer stage, tumor grade, and survival duration, was also obtained from TCGA for further analysis. Rigorous measures were taken to ensure the accuracy of the bioinformatic analysis, including the removal of duplicate patient samples.

### Exploration of NCOA7 expression across diverse cancer types

Utilizing the meticulously processed TCGA-KIRC cohort data, the study meticulously examined the expression levels of NCOA7 in ccRCC samples vis-à-vis normal samples. Visualization was accomplished using the R package ‘ggplot2,’ while statistical analyses were conducted employing the R package ‘stats.’ To assess the diagnostic efficacy of NCOA7 in ccRCC, the study employed receiver operating characteristic (ROC) curve analysis, facilitated by the ‘pROC’ R package.

Furthermore, the expression patterns of NCOA7 were explored in various cancer types, including bladder cancer, breast cancer, cervical squamous cell carcinoma, cholangiocarcinoma, and colon adenocarcinoma. Using the R package ‘dplyr,’ the patients with ccRCC were divided into two groups (NCOA7-High and NCOA7-Low) according to the expression level of NCOA7, and different clinical parameters in these two groups were calculated. Leveraging the clinical data of ccRCC patients, the study delved into the NCOA7 expression variation across different cancer stages. Additionally, the research contrasted NCOA7 expression levels among patients with distinct survival statuses.

### Prognostic significance of NCOA7 and gene set enrichment analysis

As above, patients from the TCGA-KIRC cohort were stratified into NCOA7-High and NCOA7-Low groups. Kaplan-Meier analysis, facilitated by the R packages ‘survival’ and ‘survminer,’ was conducted to assess various prognostic indicators across these patient groups. To mitigate the influence of other clinically relevant parameters, univariate and multivariate Cox regression analyses were performed using the R packages ‘survival’ and ‘rms,’ following which the nomogram and the calibration curve were also constructed. Gene Set Enrichment Analysis (GSEA) was executed to uncover potential functional implications of NCOA7, employing the ‘clusterProfiler’ R package.

### Clinical tissue specimens

Mianyang Hospital, affiliated with the School of Medicine at the University of Electronic Science and Technology, supplied 24 pairs of human ccRCC tissues and corresponding adjacent normal kidney tissues. None of the patients had undergone radiotherapy, chemotherapy, or other neoadjuvant treatments. The Ethics Committee of Mianyang Central Hospital approved the study, and informed consent was obtained from all participants (S20250212-01).

### Statistical analysis

All statistical analyses were performed using SPSS Statistics version 23.0 (IBM Corp., Chicago, IL, USA) and GraphPad Prism version 9.0 (GraphPad Software, San Diego, CA, USA). Data are presented as mean ± standard deviation (SD) from at least three independent experiments unless otherwise specified.

For comparisons between two independent groups, unpaired Student’s t-tests were used, while paired Student’s t-tests were applied for paired tissue samples. For comparisons involving more than two groups, one-way analysis of variance (ANOVA) was conducted, followed by Tukey’s post hoc test for multiple comparisons. Two-way ANOVA was employed for time-course or factorial design experiments (e.g., wound-healing assays), with appropriate post hoc analyses.

Receiver operating characteristic (ROC) curve analysis was used to evaluate the diagnostic value of NCOA7 expression, and Cox proportional hazards regression models were applied to identify survival-related risk factors. Pearson’s correlation coefficients were calculated to assess correlations between NCOA7 expression and autophagy- or lipid metabolism–related genes.

For non-normally distributed data, the Wilcoxon signed-rank test (paired samples) or the Mann–Whitney U test(independent samples) was employed. For categorical variables, the chi-square test or Fisher’s exact test was used as appropriate.

A two-sided p < 0.05 was considered statistically significant. Exact p values for all analyses are reported in the figure panels.

## Supplementary information

Supplementary Figure 1

Supplementary Figure 2

Supplementary Figure 3

Supplementary Figure 4

Supplementary Figure 5

Supplementary Figure 6

Supplementary Figure 7

Supplemengtary material

Eiditing Certificate

## Data Availability

All data supporting the findings of this study appear in the submitted manuscript or are available from the corresponding author upon reasonable request.
