# Correction: NCOA7 inhibits renal cancer progression by inducing autophagy and lipid metabolism through V-ATPase interaction

**DOI:** 10.1038/s41420-026-02952-z

**Published:** 2026-05-05

**Authors:** Jianjun Wang, Huiwen Luo, Qingliu He, Honggang Shao, Xianfu Cai, Yuan Cao, Yaodong Wang, Decai Wang

**Affiliations:** 1 Department of Hepatobiliary Surgery, Mianyang Central Hospital, School of Medicine, University of Electronic Science and Technology of China, Mianyang, China; 2 NHC Key Laboratory of Nuclear Technology Medical Transformation, Mianyang Central Hospital, School of Medicine, University of Electronic Science and Technology of China, Mianyang, China; 3 Department of Urology, The Second Affiliated Hospital of Fujian Medical University, Quanzhou, China; 4 Department of Urology, Mianyang Central Hospital, School of Medicine, University of Electronic Science and Technology of China, Mianyang, China

**Keywords:** Urological cancer, Cancer

Correction to: *Cell Death Discovery* 10.1038/s41420-025-02766-5, published online 21 October 2025

In this article the author Yaodong Wang has been listed as a co-corresponding author.

The original article has been corrected.

