# Supplementary figures and images for: NCOA7 inhibits renal cancer progression by inducing autophagy and lipid metabolism through V-ATPase interaction
Source: Cell Death Discov. 2025 Oct 21;11:471. doi: 10.1038/s41420-025-02766-5 (PMC12540685; doi:10.1038/s41420-025-02766-5)

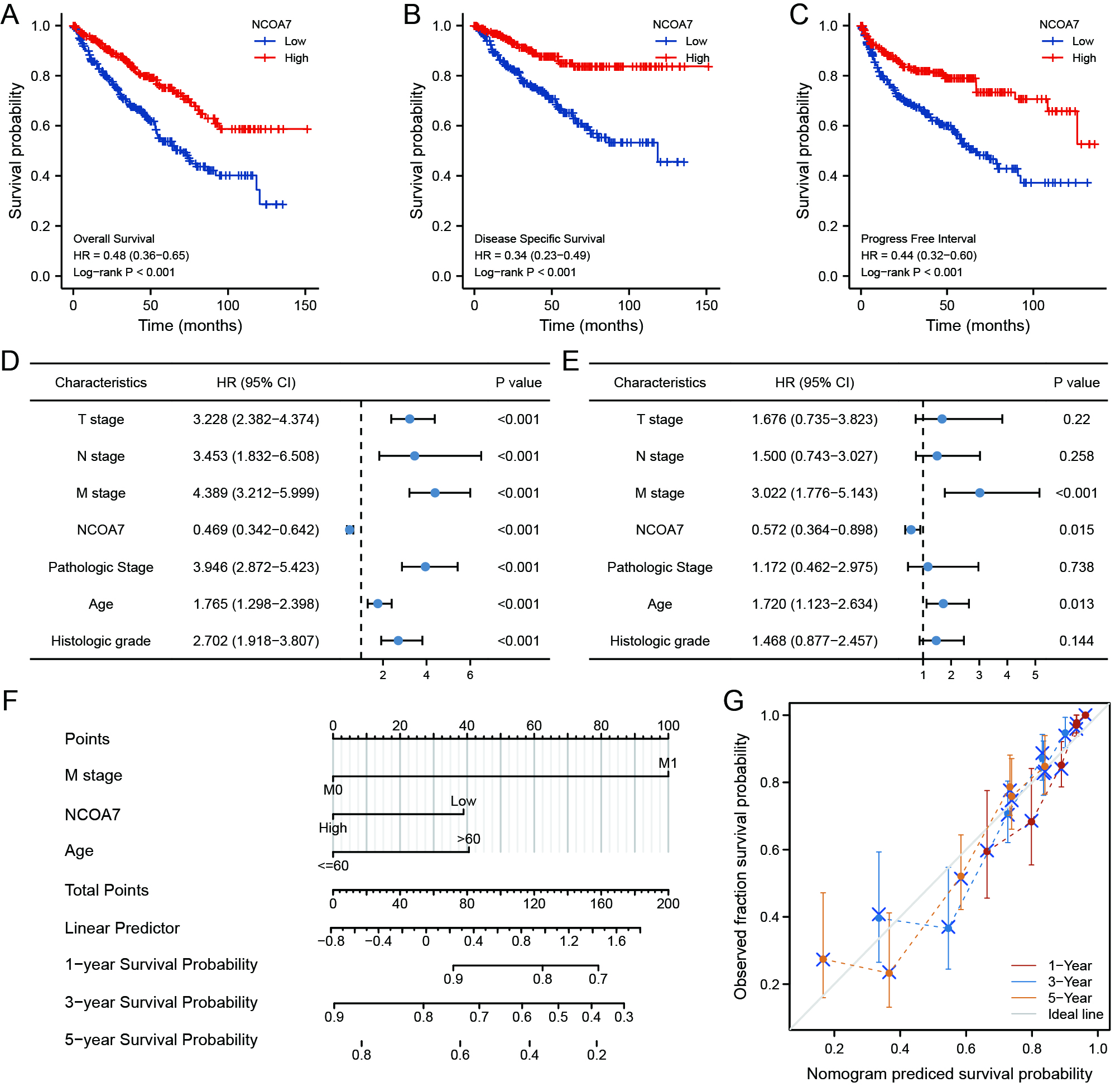

Supplement: Supplementary file 1 — Supplementary Figure 1 [file 41420_2025_2766_MOESM1_ESM.jpg]

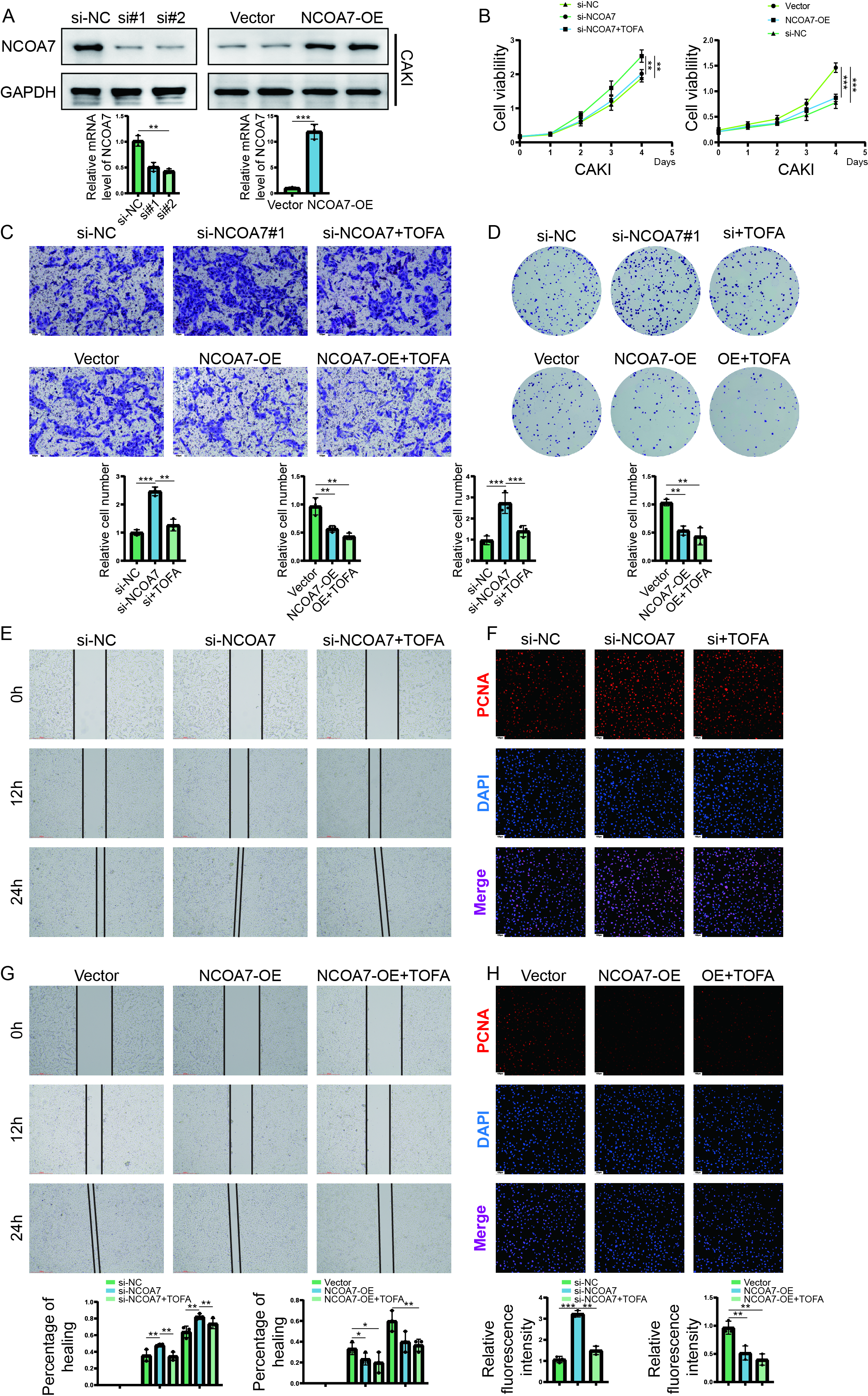

Supplement: Supplementary file 2 — Supplementary Figure 2 [file 41420_2025_2766_MOESM2_ESM.jpg]

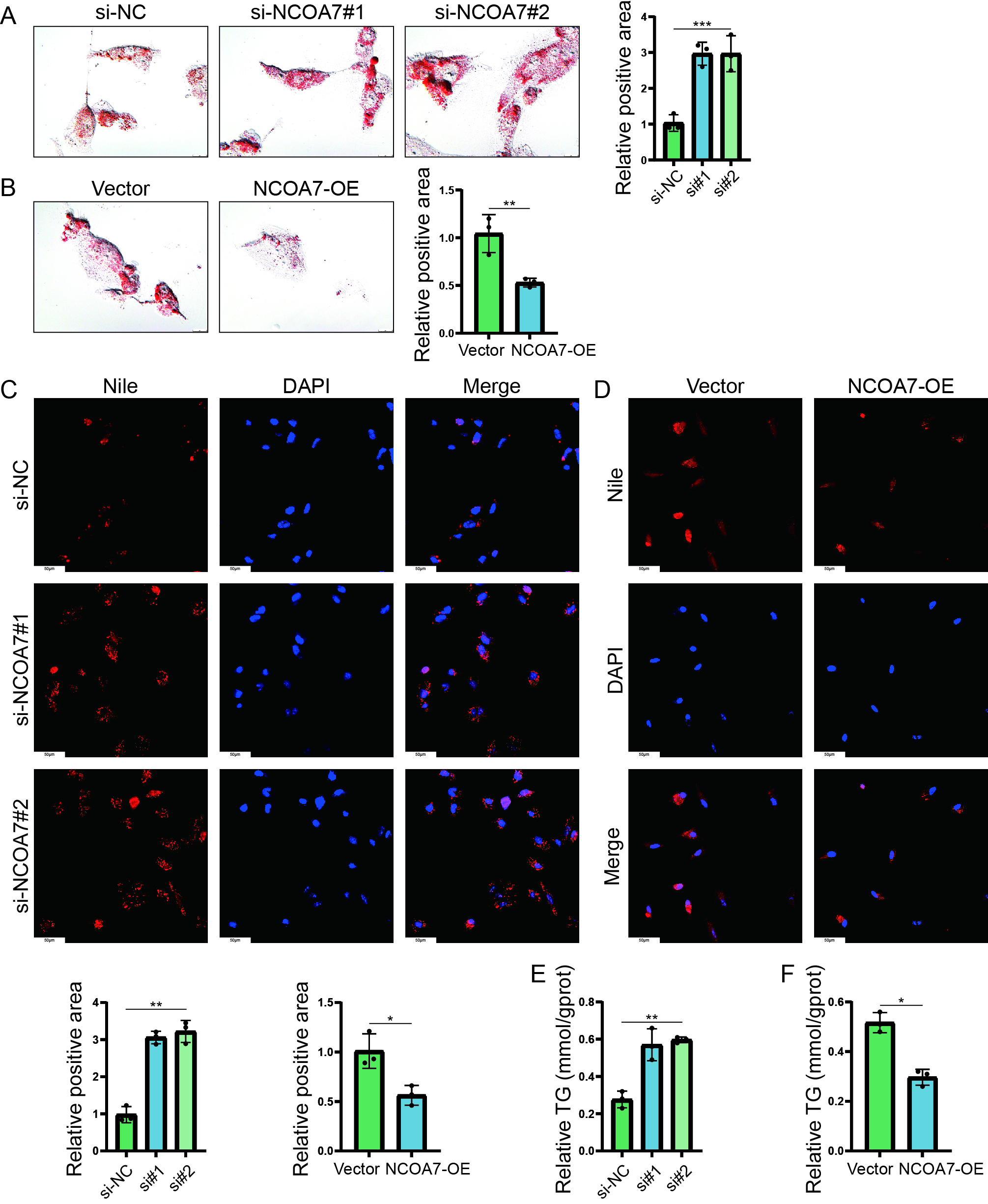

Supplement: Supplementary file 3 — Supplementary Figure 3 [file 41420_2025_2766_MOESM3_ESM.jpg]

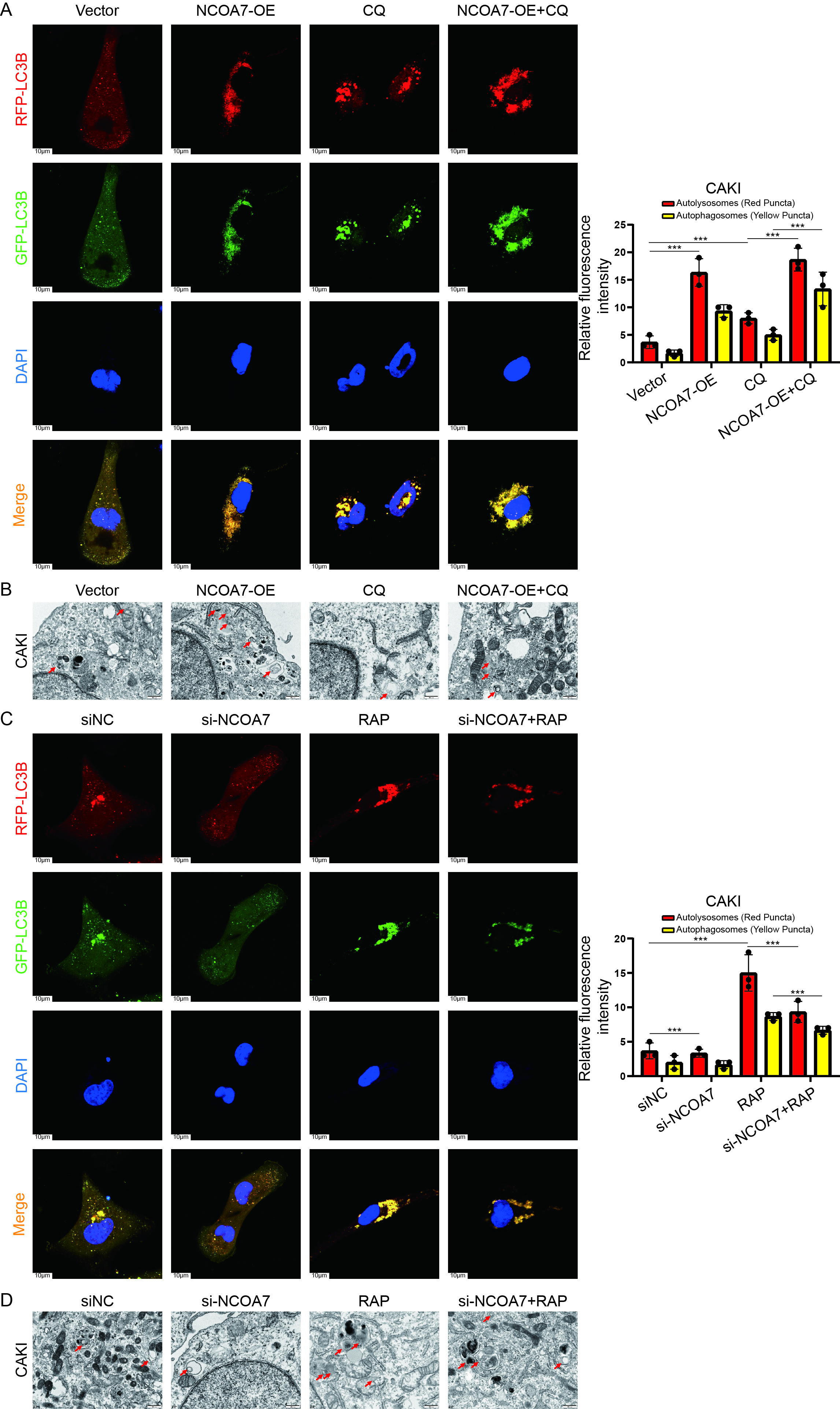

Supplement: Supplementary file 4 — Supplementary Figure 4 [file 41420_2025_2766_MOESM4_ESM.jpg]

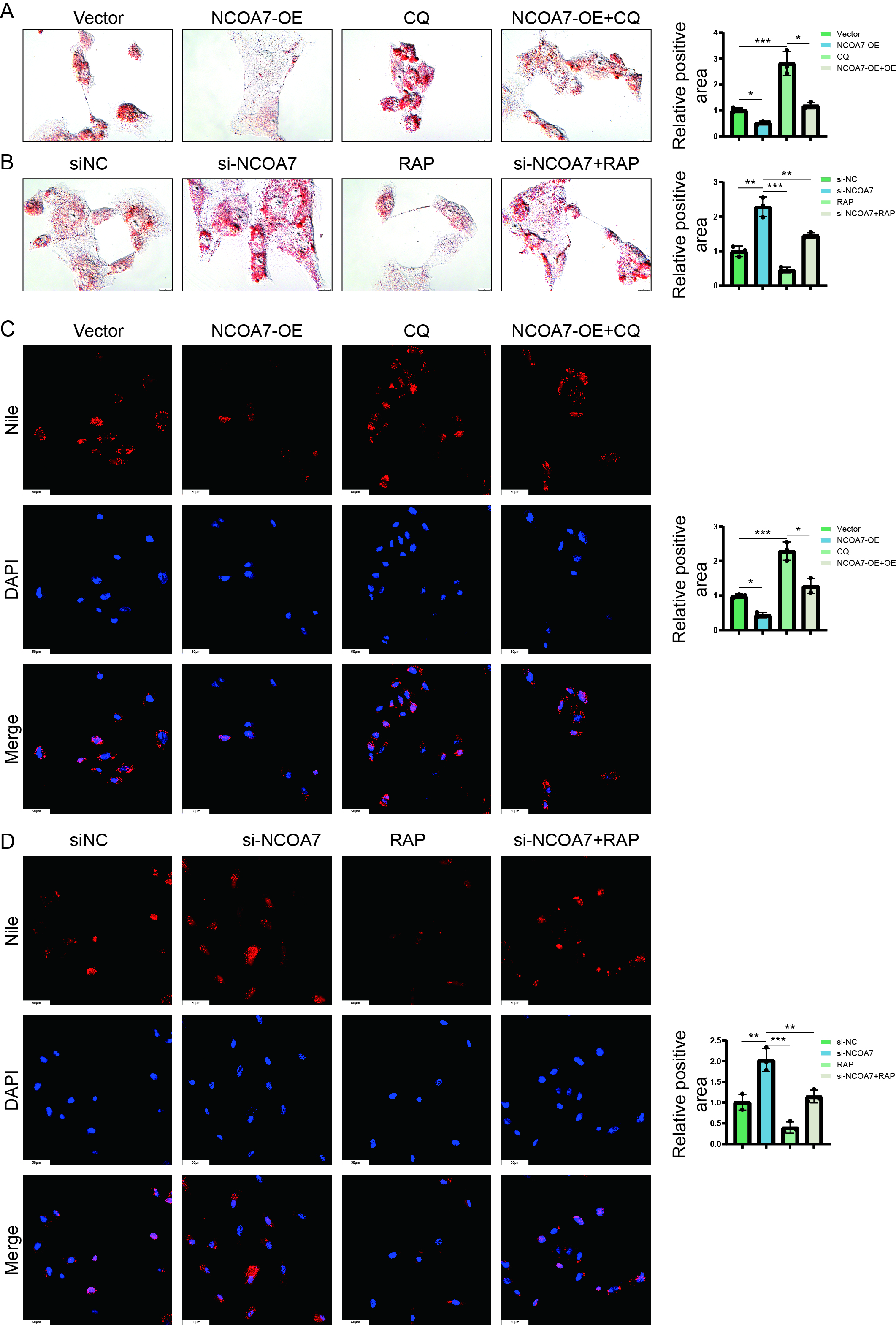

Supplement: Supplementary file 5 — Supplementary Figure 5 [file 41420_2025_2766_MOESM5_ESM.jpg]

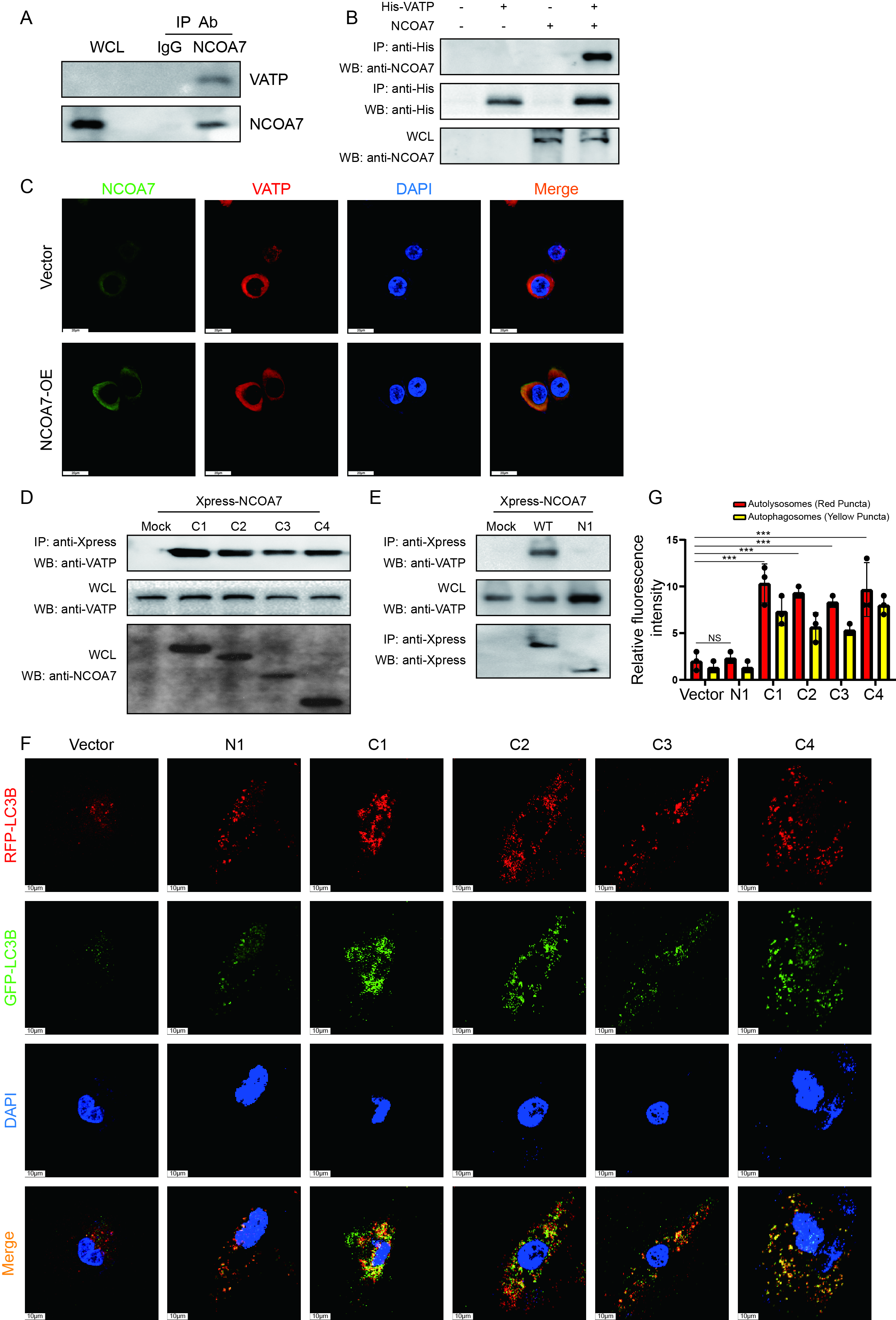

Supplement: Supplementary file 6 — Supplementary Figure 6 [file 41420_2025_2766_MOESM6_ESM.jpg]

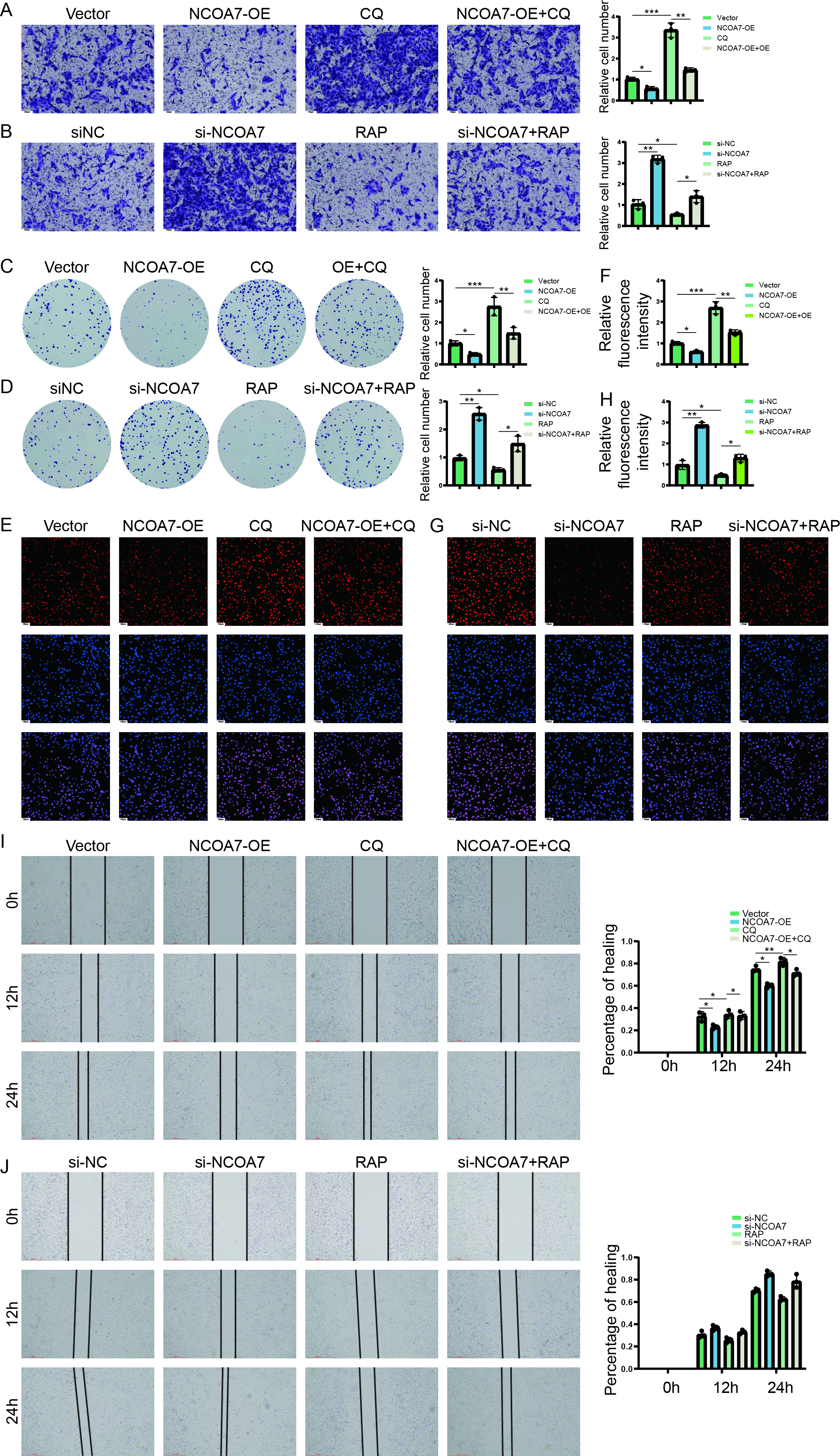

Supplement: Supplementary file 7 — Supplementary Figure 7 [file 41420_2025_2766_MOESM7_ESM.jpg]

Figure 3

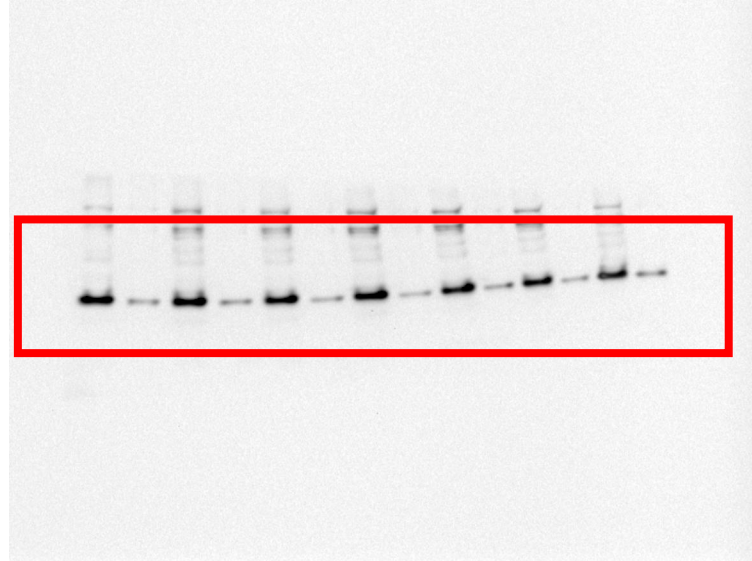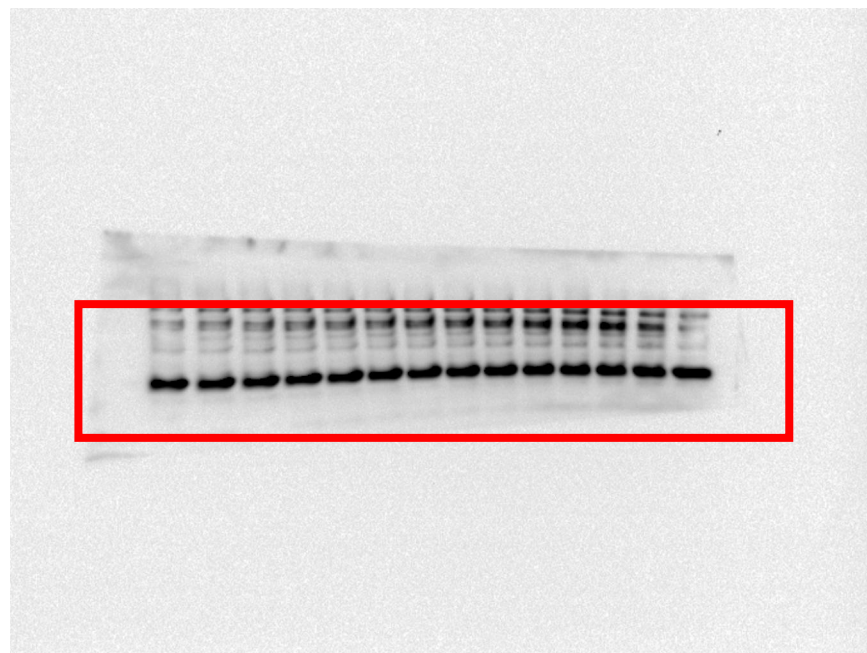

Figure 3

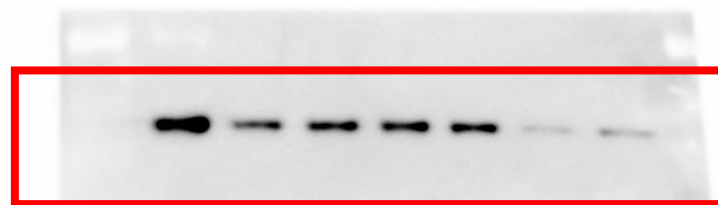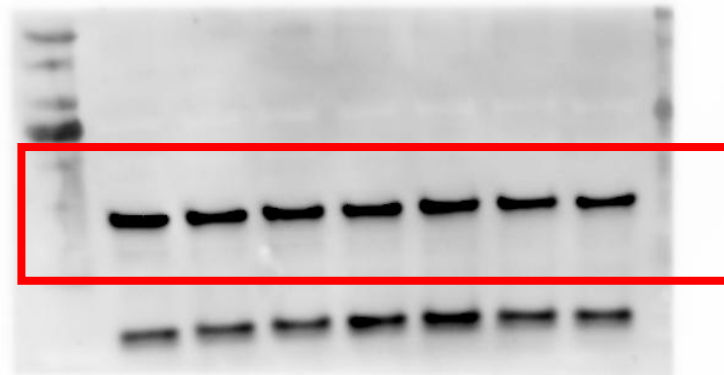

Figure 4

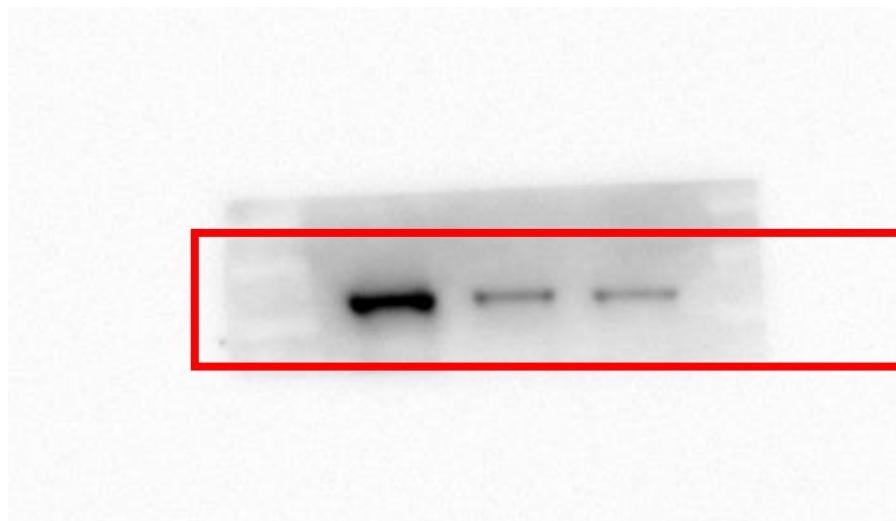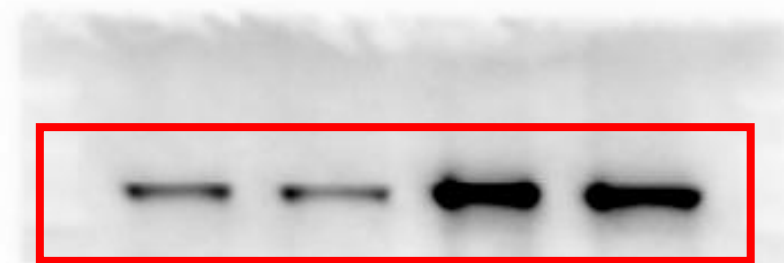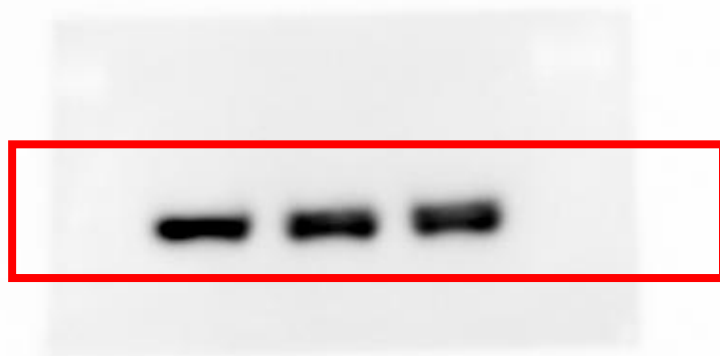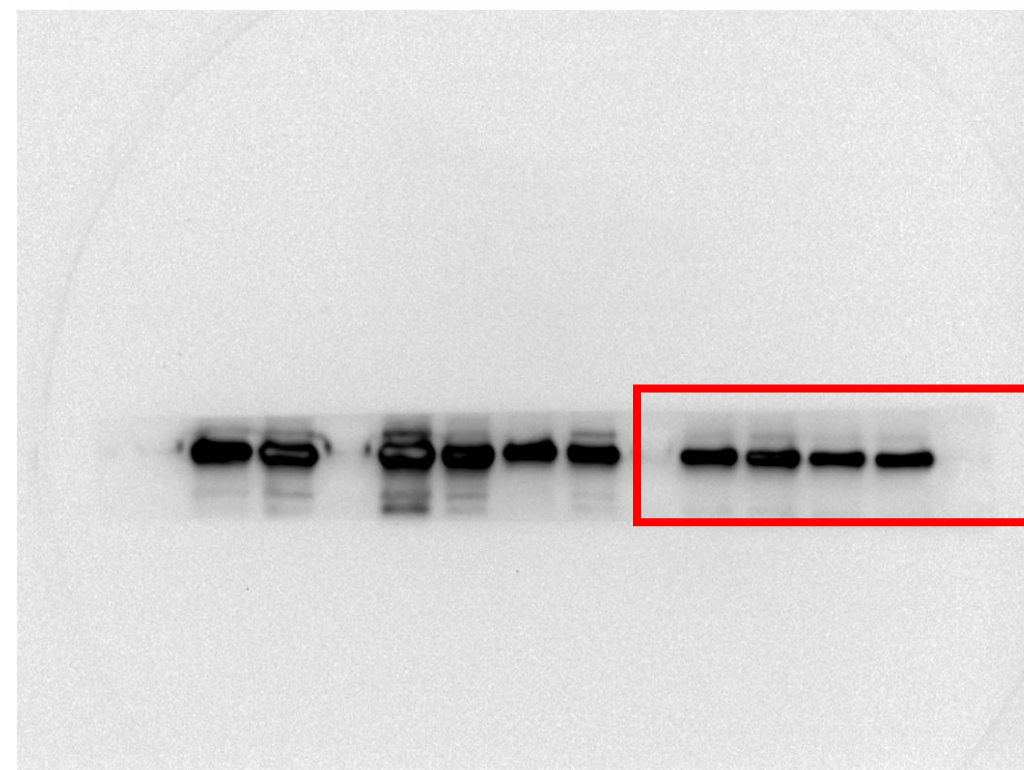

Figure 6

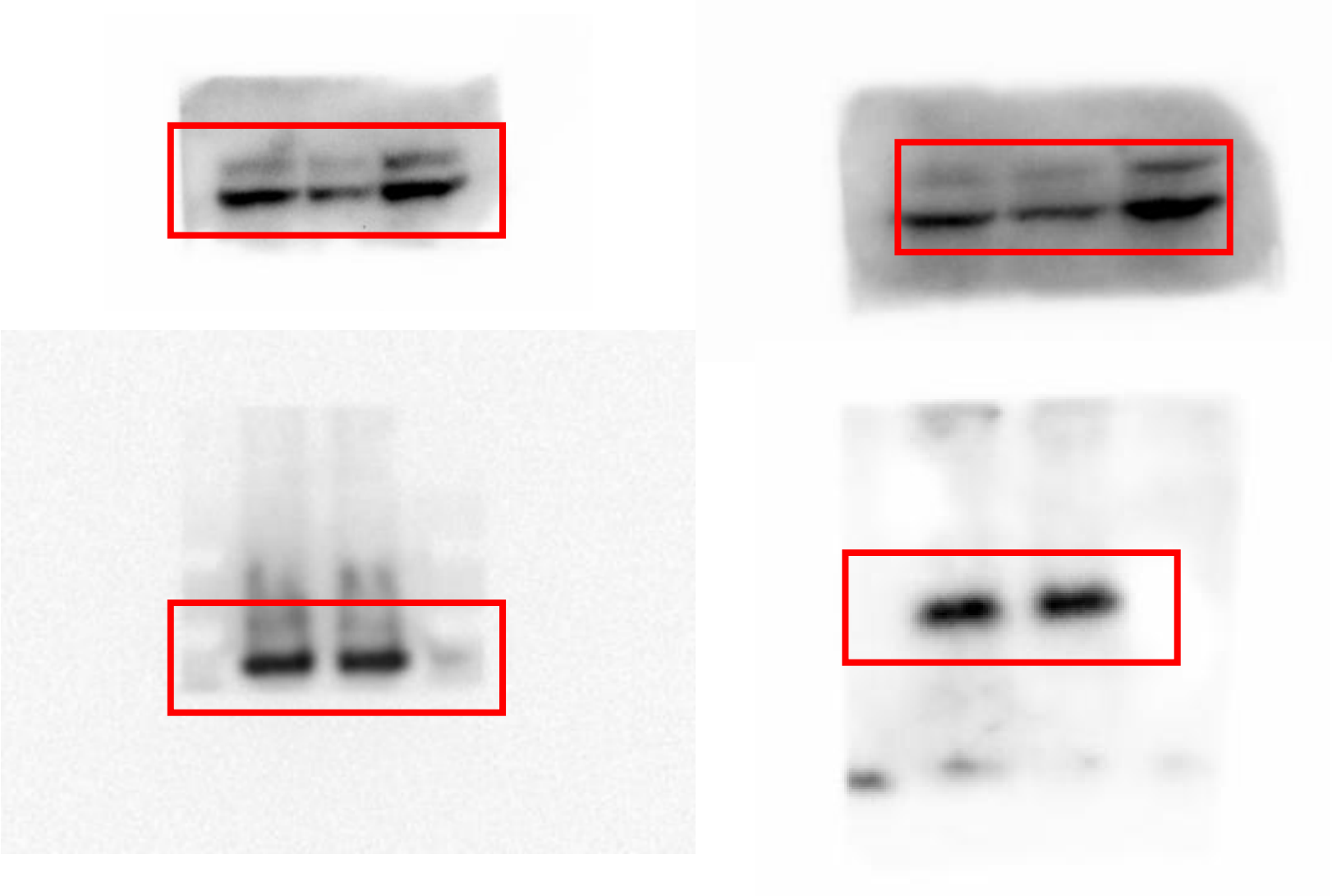

Figure 8

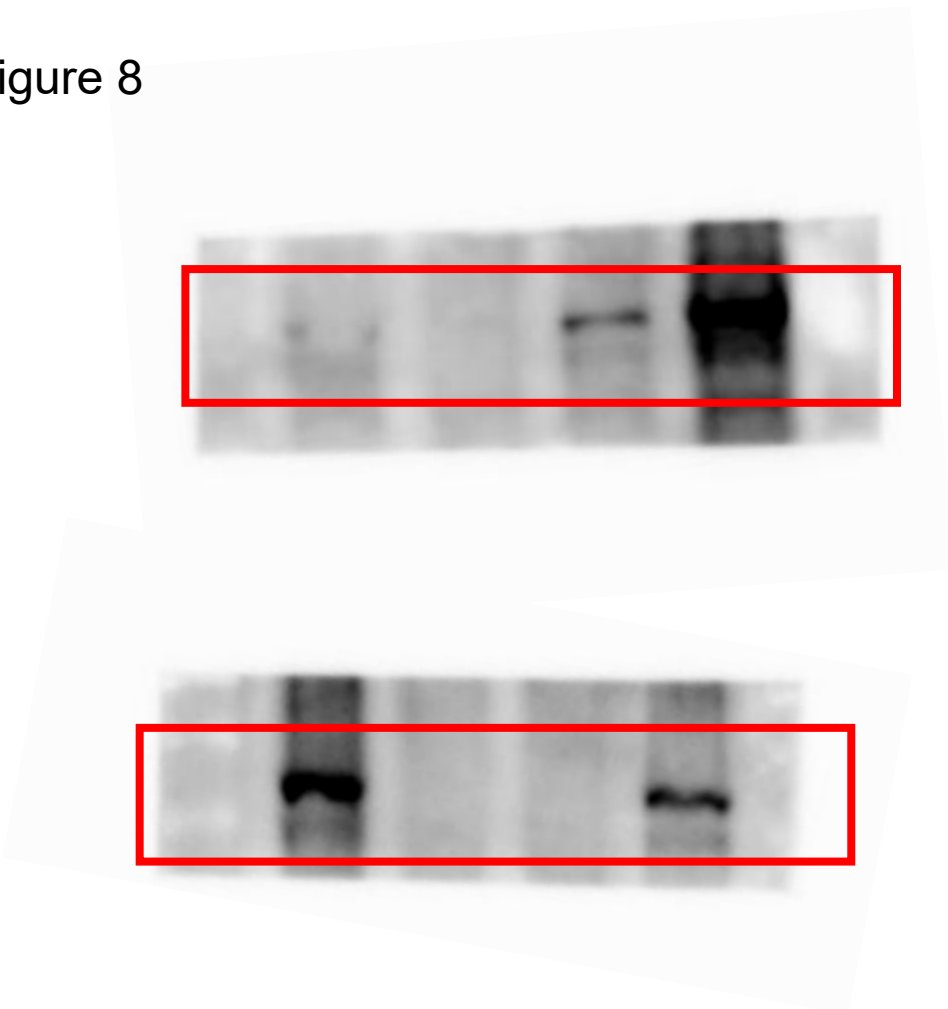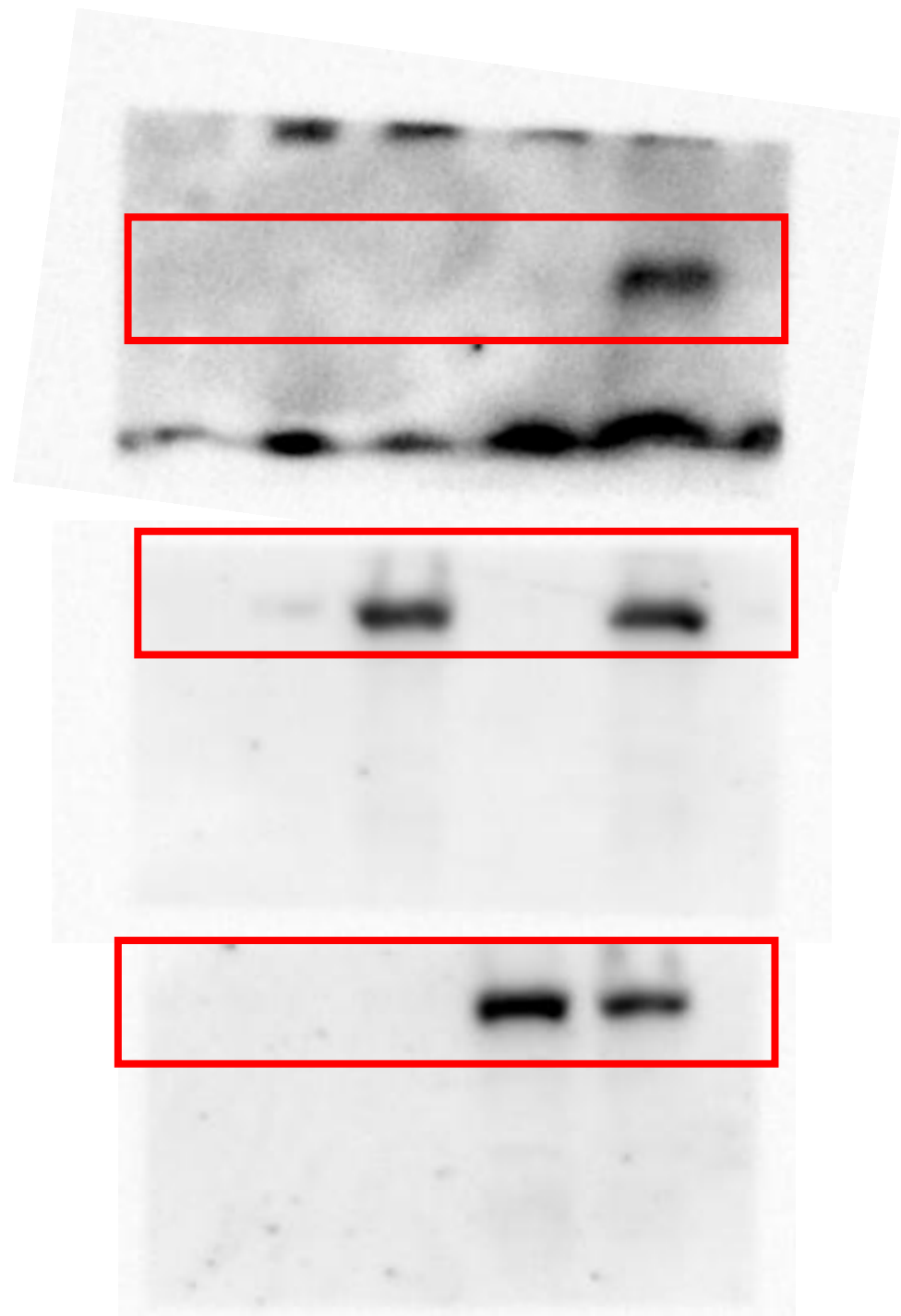

Figure 8

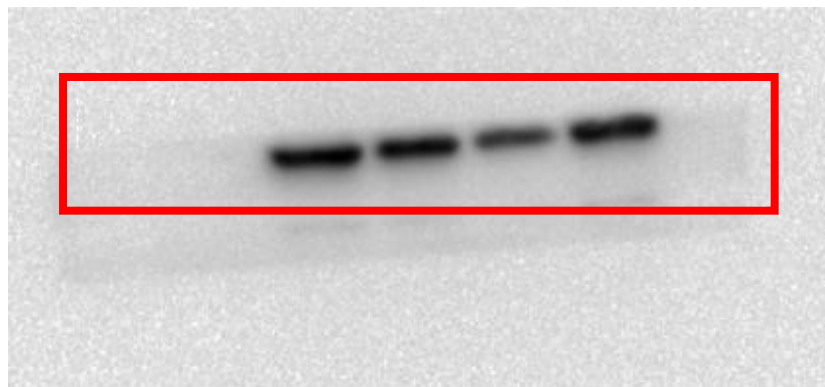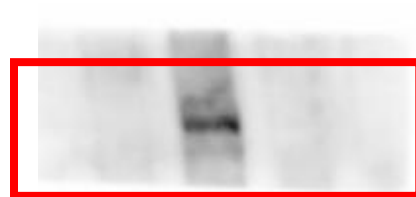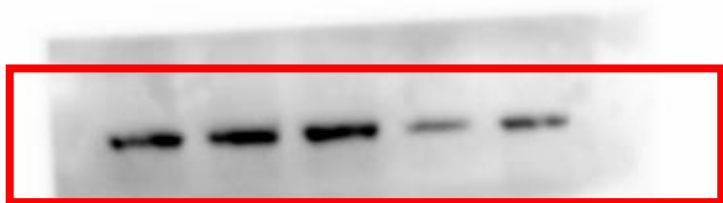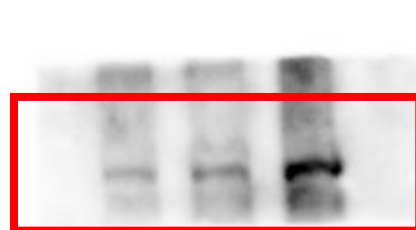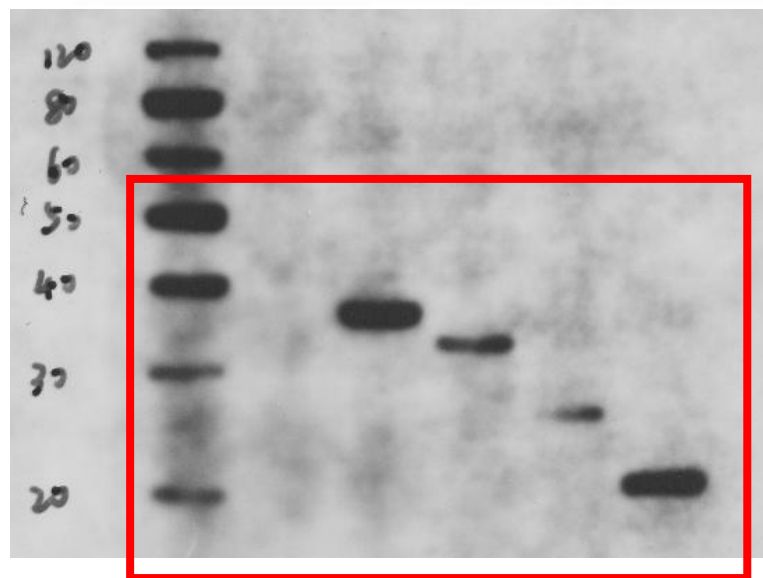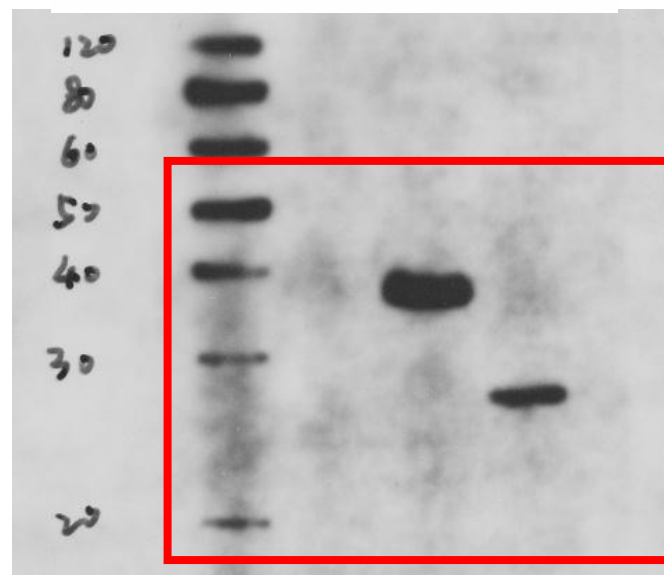

sFigure 1

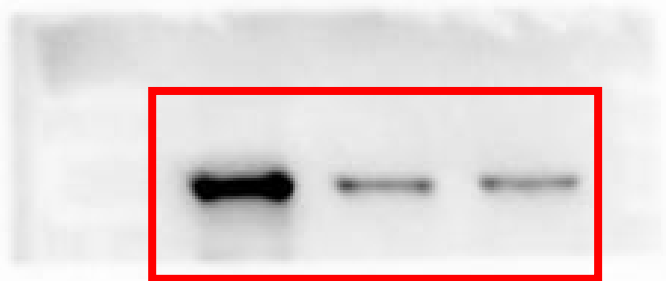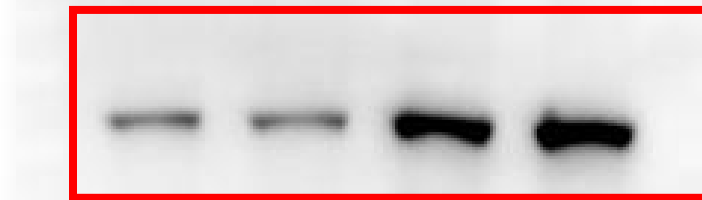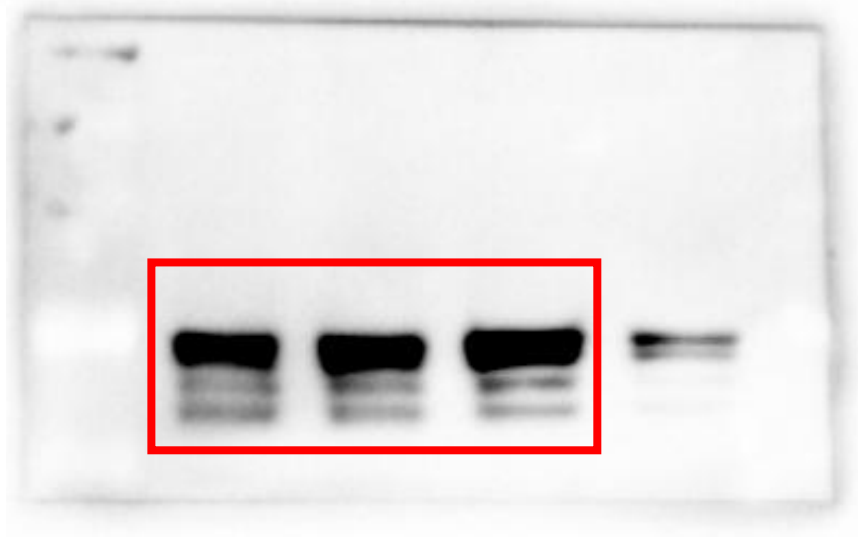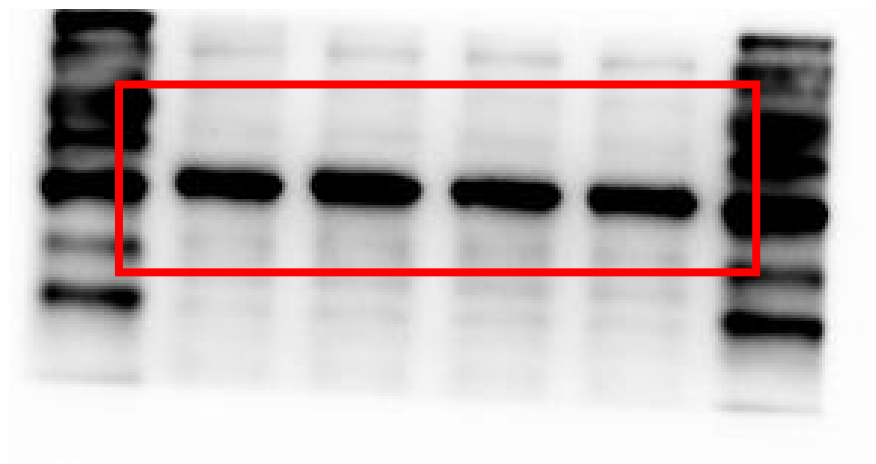

sFigure 5

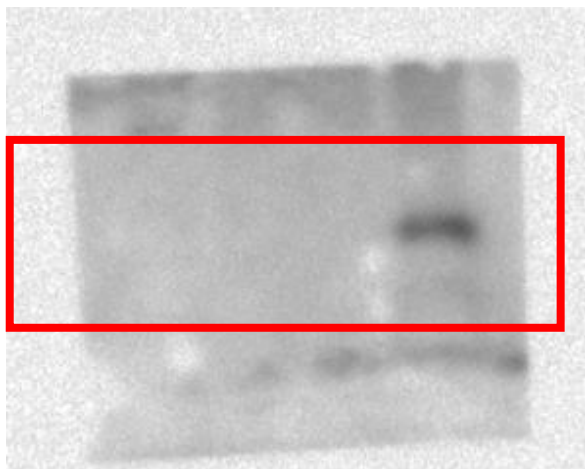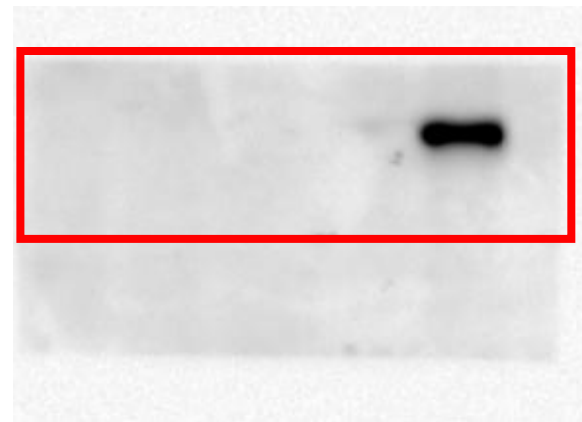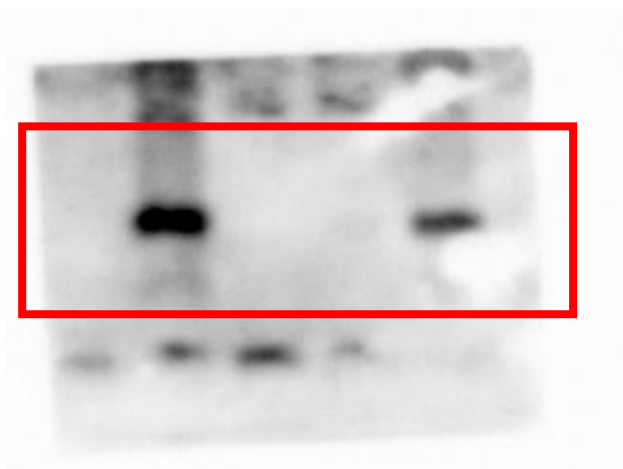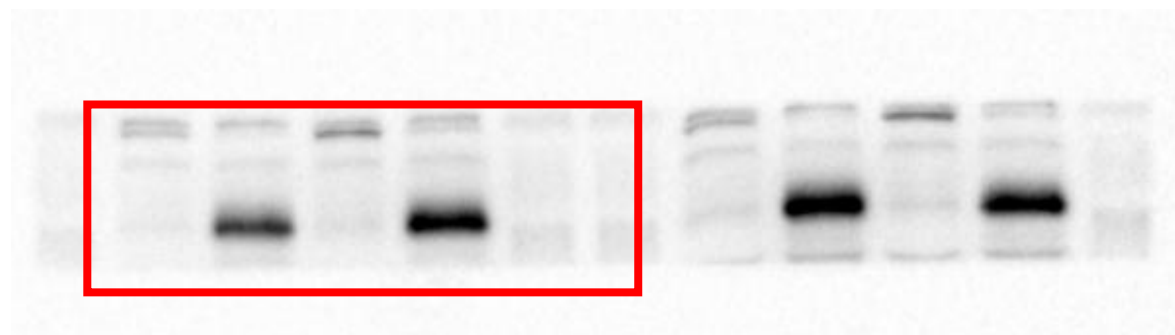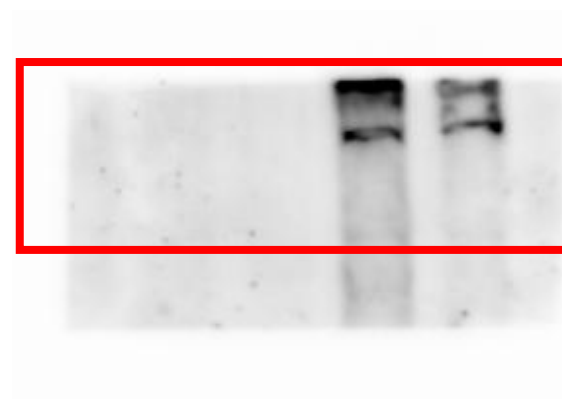

sFigure5

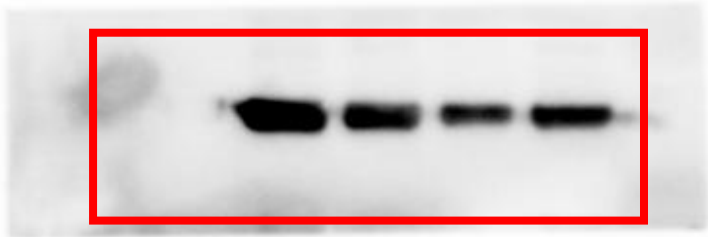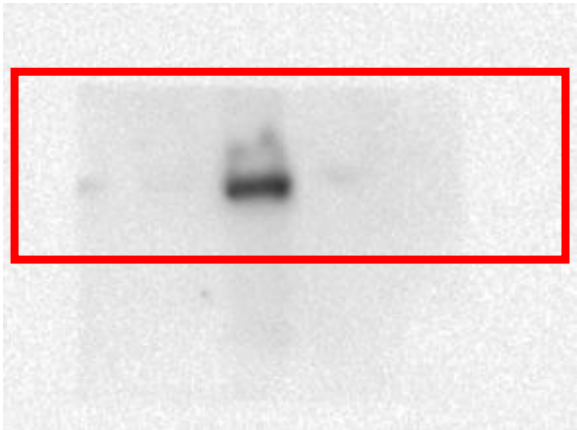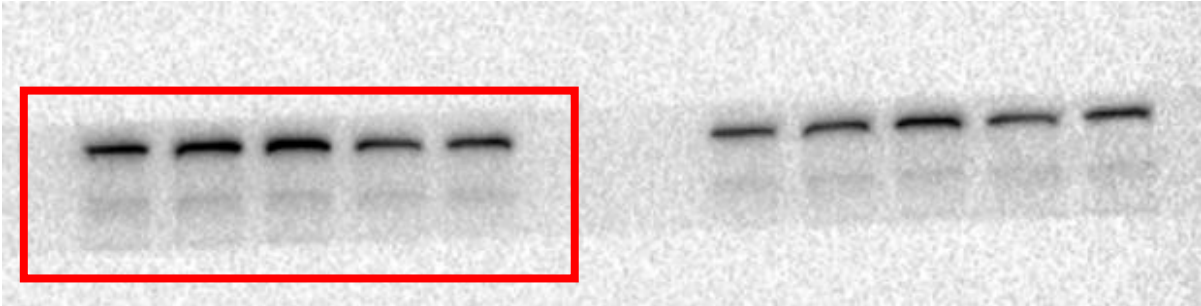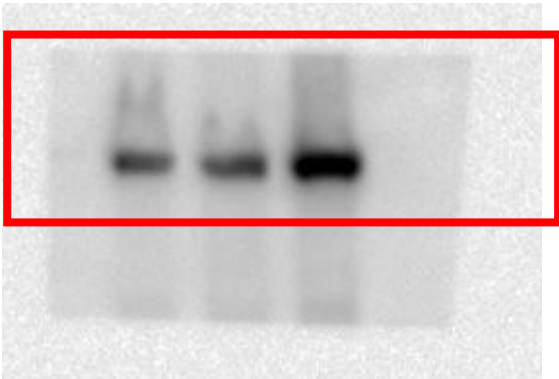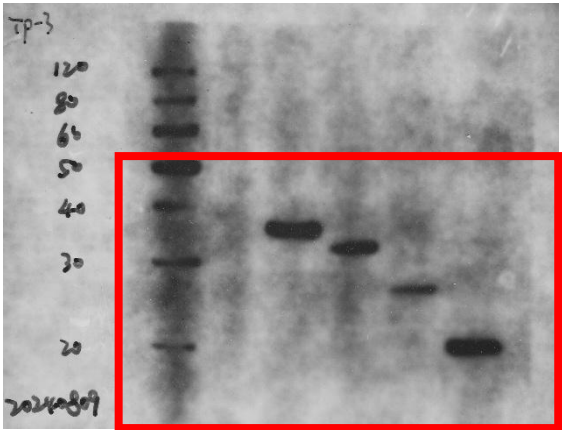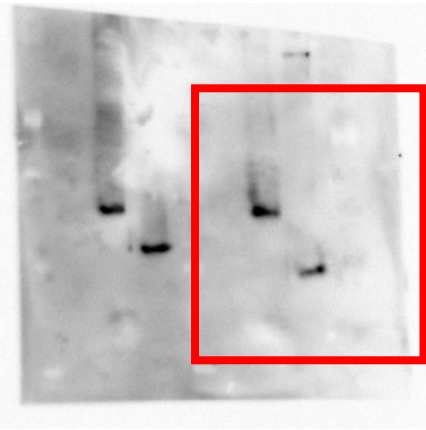

Supplement: Supplementary file 9 — Eiditing Certificate [file 41420_2025_2766_MOESM9_ESM.pdf]
