# Supplementary material for: NCOA7 inhibits renal cancer progression by inducing autophagy and lipid metabolism through V-ATPase interaction
Source: Cell Death Discov. 2025 Oct 21;11:471. doi: 10.1038/s41420-025-02766-5 (PMC12540685; doi:10.1038/s41420-025-02766-5)
Supplement: Supplementary file 8 — Supplemengtary material [file 41420_2025_2766_MOESM8_ESM.docx]

2.1 Cell culture

The Cell Bank of the Chinese Academy of Sciences provided human ccRCC cell lines (A498, 786-O, CAKI, OSRC, SN12-PM6 and ACHN) as well as the human renal tubular epithelial cell line (HK-2). All cell cultures were maintained in a medium containing 10% fetal bovine serum (FBS; Invitrogen, Thermo Fisher Scientific, Waltham, MA, USA) and 1% antibiotics (Biosharp, Beijing, China) under humidified conditions at 37°C.

2.2 Transfection of lentiviral plasmids

Lentiviral plasmids for NCOA7 overexpression and corresponding control vectors were sourced from Shanghai GeneChem (Shanghai, China). The transfection reagent Lipofectamine 3000 was obtained from Invitrogen (Thermo Fisher Scientific, USA). Cells (786-O, CAKI) were seeded in 6-well plates at 70% confluence and allowed to adhere for 24 hours. Transfection complexes were created by combining lentivirus with 5 μL of Lipofectamine 3000, incubating the mixture at room temperature for 15 minutes, and subsequently adding it to the cells. After 24 hours of infection, the medium was replaced with fresh medium containing 3 μg/mL puromycin. The cells were then cultured for an additional 1–2 weeks to select stably infected cell lines. An empty vector served as the control.

2.3 Lentiviral transduction

Cells were grown in 6-well plates until they reached 30%-40% confluence, at which point the medium was replaced with serum-free medium. For viral transduction, 40 μL each of transfection reagent A and transfection reagent P (both from Shanghai Genechem, China) were added, along with lentivirus at a multiplicity of infection of 40. After 8–10 hours, the medium was replaced with fresh medium containing 10% serum. Cells were cultured for an additional 3 days before undergoing puromycin selection to establish stable cell lines.

2.4 Cell viability assay

The cell viability of 786-O and CAKI cells was determined using a Cell Counting Kit-8 (CCK-8) assay (Biosharp, Beijing, China). In a 96-well plate, each well was seeded with 3 × 10^3 cells (786-O and CAKI) and incubated for 0, 1, 2, 3, or 4 days. Following incubation, 10 μL of CCK-8 solution was added to each well, and the cells were incubated for an additional 30 minutes. Absorbance at 450 nm was then measured using a multifunctional enzyme marker (PerkinElmer, Waltham, MA, USA).

2.5 Colony formation assay

A colony formation assay was conducted to investigate the impact of NCOA7 on the proliferation of ccRCC cells. 786-O and CAKI cells (700 cells per well) were plated in 6-well plates and cultured for 14 days. Afterward, the cells were fixed with 4% paraformaldehyde and stained with 0.1% crystal violet solution at room temperature. Once the plates were dried, colonies were counted to assess and compare the colony-forming ability under various experimental conditions.

2.6 Wound healing assay

786-O and CAKI cells were plated in 6-well plates and cultured until they reached 80% confluence. A sterile 10 μL pipette tip was used to create a straight scratch across the cell monolayers. The wells were then washed three times with phosphate-buffered saline (PBS) and incubated in medium containing 10% FBS for 24 hours. Wound widths were captured using a light microscope (Olympus, Japan) at 0, 12, and 24 hours, and the images were analyzed with ImageJ software.

2.7 Transwell invasion assay

The Transwell assay (BD Biosciences, Franklin Lakes, NJ, USA) was employed to evaluate the impact of NCOA7 expression levels on the invasive capability of ccRCC cells. 200 μL of 786-O and CAKI cells (3 × 10^4 cells per well) in serum-free medium were added to the upper chamber, while the lower chamber was filled with 700 μL of complete medium containing 20% FBS. After 24 hours of incubation, the cells in the lower chamber were fixed with 4% paraformaldehyde for 30 minutes and stained with 0.1% crystal violet for 20 minutes. The migrated cells were then washed with PBS (Biosharp, Beijing, China) and observed using an inverted microscope (Olympus, Japan).

2.8 Protein blotting

Proteins were extracted from tumor tissues or cells using RIPA protein lysis buffer supplemented with a protease inhibitor mixture and phenylmethylsulfonyl fluoride (PMSF). Protein concentrations were determined three times for each sample using the BCA method. Each sample (40 micrograms of protein) was subjected to gel electrophoresis, and the separated proteins were transferred onto a polyvinylidene difluoride (PVDF) membrane. The membranes were then blocked with 5% milk for 1 hour at room temperature, followed by overnight incubation with primary antibodies. After three washes with PBS, the membranes were incubated with secondary antibodies for 1 hour at room temperature. Protein bands were visualized using a ChemiDoc MP imaging system (Bio-Rad, Hercules, CA, USA). The antibodies used included: NCOA7 (Proteintech, 23092-1-AP), LC3 (Proteintech, 14600-1-AP), ATG5 (Proteintech, A7553), SQSTM1 (Proteintech, 18420-1-AP), and anti-GAPDH (Proteintech, 60004-1-Ig).

2.9 Immunofluorescence staining

Cell slides were fixed with 4% paraformaldehyde and then treated with goat serum albumin. Primary antibody PCNA (1:100, Proteintech, 10205-2-AP) was added and incubated overnight at 4°C. The slides were subsequently incubated with fluorescently labeled secondary antibodies for 1 hour at room temperature. Cell nuclei were stained with DAPI for 10 minutes, and images were captured using a confocal microscope (Leica, Germany).

2.10 Tissue immunohistochemistry (IHC) and immunofluorescence staining

The tissue specimens were embedded in paraffin and sectioned. Dewaxing and rehydration were performed using EDTA. Antigen retrieval was achieved by incubating the sections at 120°C for 5 min. The sections were then treated with 3% H_2_O_2_ for 15 min at room temperature to block endogenous peroxidase activity. After serum blocking, tissue sections were incubated overnight at 4°C using primary antibody. Immunodetection was performed the following day using secondary antibodies, and the results were visualized using DAB. Sections were then stained with hematoxylin. Primary antibodies used in IHC included those used for protein blotting, with additional antibody information as follows: ki67 (Abclonal, A2094).

For tissue immunofluorescence staining, specimens were fixed with 4% paraformaldehyde and treated with Triton X. The samples were then sealed in 5% goat serum. The cells were then incubated with the primary antibody. Nuclei were stained with DAPI and immunodetected using a fluorescent secondary antibody.

2.11 Cell immunofluorescence and confocal microscopy

The cells were fixed with 4% paraformaldehyde. For immunostaining, they were first blocked with 10% goat serum (Gibco, 16210) in PBS (Wellgene, ML008) and then incubated with a 1:500 dilution of primary antibody in PBS. Subsequently, the cells were stained with a 1:1000 dilution of fluorescence-conjugated secondary antibody (Invitrogen). The slides were washed three times with PBS, and the nuclei were stained with DAPI. Images were captured using a confocal microscope (Leica, Germany).

2.12 RNA isolation and real-time fluorescence quantitative PCR analysis

RNA was extracted using the TRIzol reagent (Thermo Fisher Scientific, Waltham, MA, USA). The concentration and purity of the RNA were measured with a NanoDrop 2000 spectrophotometer (NanoDrop Technologies, Wilmington, DE, USA). One microgram of RNA was used for reverse transcription. PCR was conducted using the SYBR Green mixture (YEASEN, China) on a StepOnePlus real-time fluorescence quantitative PCR system (Thermo Fisher Scientific). Primers for miRNAs were sourced from RuiBo Bio. The gene primers used are listed below：gene primers NCOA7 (forward, 5’-GCACAGGCGAAACTTTTCTCT-3’, reverse, 5’-AGCCATAAACCAAATCGTCCC-3’), and GAPDH (forward, 5’-GAGTCAACGGATTTGGTCGT-3’, reverse, 5’-GACAAGCTTCCCGTTCTCAG-3’).

2.13 Oil Red O Staining

Cells were cultured in confocal Petri dishes until they reached 30% confluence. An Oil Red O working solution was prepared by mixing saturated Oil Red O and ultrapure water in a 2:3 ratio. After removing the cell culture medium, the cells were washed twice with PBS and then fixed with 4% paraformaldehyde. The Oil Red O solution was added to stain the cells for 30 minutes at room temperature. The cells were subsequently washed, dried, and imaged using a confocal microscope.

2.14 Nile Red staining

After fixation with 4% paraformaldehyde, a freshly prepared 1X Nile Red staining working solution (Beyotime) was added to the cells and incubated for 10–20 minutes at room temperature in the dark. After washing with PBS, the nuclei were stained with Hoechst 33342. Images were then acquired using confocal microscopy.

2.15 Triglyceride assay

Cells were cultured in 10 cm dishes and prepared as cell suspensions. The cells were centrifuged at 1000 rpm for 10 min; the supernatant was discarded, and the cell pellet was retained. Cells were lysed using 2% TritonX-100 for 40 min. Then, 2.5 μL of the lysate was added to 250 μL of working solution and incubated at 37°C for 10 min. A portion of the sample was used for protein quantification. The absorbance was measured at 510 nm using an enzyme marker. The triglyceride content was calculated using the following formula:

Triglyceride content = (Sample OD-Blank OD)÷(Calibration OD-Blank OD) × Standard concentration÷Protein concentration

2.16 Immunoprecipitation (Co-IP) assay

Approximately 10^7^ cells were harvested and lysed in IP buffer containing 50 mM Tris-HCl (pH 7.4), 150 mM NaCl, 2 mM EDTA, and 1% NP-40 for 30 minutes. The lysate supernatants were then incubated with the primary antibody for 2 hours. Protein A/G beads were added to each sample afterward. Unbound materials were washed away, and the magnetic beads were resuspended in 2× sample buffer and heated to 95°C for 10 minutes. The following antibodies were utilized for the Co-IP assay.

2.17 LC-MS Analysis of Lipid and Cholesterol Metabolism in 786-O Renal Cancer Cells

For targeted lipidomics and cholesterol metabolic profiling, human renal carcinoma 786-O cells were cultured, treated as indicated, and harvested at a density of 1 × 10⁶ cells per 16 × 100 mm glass tube (Pyrex; 9826). To release the free sterol pool, cells were hydrolyzed by the addition of 1 mL of 0.5 M KOH in methanol, followed by incubation at 80°C for 1 hour.

After hydrolysis, sterols and lipid metabolites were extracted using a modified Bligh and Dyer liquid-liquid extraction protocol. Briefly, 2 mL of dichloromethane and 1 mL of water were added to each sample. The mixture was vortexed thoroughly and centrifuged to allow phase separation. The lower organic phase, containing the lipid fraction, was carefully collected and evaporated to dryness under a gentle stream of nitrogen gas.

The dried extracts were reconstituted in 300 µL of 90% methanol and subjected to liquid chromatography–mass spectrometry (LC-MS) analysis. Lipid species were separated and analyzed using a SCIEX API 5000 triple quadrupole mass spectrometer coupled to a Shimadzu LC-30AD HPLC system. Chromatographic separation was performed on a Kinetex C18 HPLC column (150 × 2.1 mm, 2.6 µm particle size; Phenomenex).

Data acquisition and quantitative analysis were conducted using MultiQuant software (SCIEX). Lipid and cholesterol metabolites were identified and quantified based on retention time and mass-to-charge ratio (m/z) compared to internal standards and authenticated reference compounds.

2.18 In vivo tumor implantation

The experimental animals were divided into two groups, with five animals in each group: experimental and control. Tumor cells (2×10^6^) were subcutaneously injected into nude mice. The metastatic potential of the tumor cells was evaluated via tail vein injection. Tumor size was recorded every five days, with the final measurement taken on day 40. After collecting tissue samples, immunohistochemical staining was performed following previously established methods. All animal experiments were conducted with the approval of the Professional Committee for the Use and Care of Animals at Mianyang Hospital, School of Medicine, University of Electronic Science and Technology.
